# A comprehensive characterization of the caspase gene family in insects from the order Lepidoptera

**DOI:** 10.1186/1471-2164-12-357

**Published:** 2011-07-08

**Authors:** Juliette Courtiade, Yannick Pauchet, Heiko Vogel, David G Heckel

**Affiliations:** 1 Department of Entomology, Max Planck Institute for Chemical Ecology, Hans-Knöll-Str. 8, 07745 Jena, Germany

## Abstract

**Background:**

The cell suicide pathway of apoptosis is a necessary event in the life of multicellular organisms. It is involved in many biological processes ranging from development to the immune response. Evolutionarily conserved proteases, called caspases, play a central role in regulating apoptosis. Reception of death stimuli triggers the activation of initiator caspases, which in turn activate the effector caspases. In Lepidoptera, apoptosis is crucial in processes such as metamorphosis or defending against baculovirus infection. The discovery of p35, a baculovirus protein inhibiting caspase activity, has led to the characterization of the first lepidopteran caspase, Sf-Caspase-1. Studies on Sf-Caspase-1 mode of activation suggested that apoptosis in Lepidoptera requires a cascade of caspase activation, as demonstrated in many other species.

**Results:**

In order to get insights into this gene family in Lepidoptera, we performed an extensive survey of lepidopteran-derived EST datasets. We identified 66 sequences distributed among 27 species encoding putative caspases. Phylogenetic analyses showed that Lepidoptera possess at least 5 caspases, for which we propose a unified nomenclature. According to homology to their *Drosophila *counterparts and their primary structure, we determined that Lep-Caspase-1, -2 and -3 are putative effector caspases, whereas Lep-Caspase-5 and -6 are putative initiators. The likely function of Lep-Caspase-4 remains unclear. Lep-Caspase-2 is absent from the silkworm genome and appears to be noctuid-specific, and to have arisen from a tandem duplication of the Caspase-1 gene. In the tobacco hawkmoth, 3 distinct transcripts encoding putative Caspase-4 were identified, suggesting at least 2 duplication events in this species.

**Conclusions:**

The basic repertoire of five major types of caspases shared among Lepidoptera seems to be smaller than for most other groups studied to date, but gene duplication still plays a role in lineage-specific increases in diversity, just as in Diptera and mammals.

## Background

Apoptosis, a distinctive and highly regulated type of cell suicide, is fundamental for various biological processes such as development [1], tissue homeostasis, DNA damage response [2] and immune response [3]. Development of holometabolous insects is characterized by a complete metamorphosis between the wingless larval stage, mostly dedicated to nutrient acquisition and growth, and the winged adult form, dedicated to reproduction. These drastic modifications in appearance and physiology require massive histolysis and histogenesis. The importance of apoptotic events during development of holometabolous insects and more specifically in Lepidoptera has been shown as early as the 1960's. In wild silkmoths and the tobacco hawkmoth, the first ecdysone peak during metamorphosis induces apoptotic degeneration of the larval intersegmental muscles, proleg motoneurons, and labial glands [4-6]. The decrease in ecdysone titer shortly before hatching induces apoptotic degeneration of abdominal neurons and intersegmental muscles [7]. Important changes in food habits between larval and adult stages imply extensive remodeling of the digestive tract. For example, in *Galleria mellonella*, the larval midgut undergoes apoptosis during metamorphosis [8]. Similarly, in *Heliothis virescens*, apoptosis of the larval midgut has been correlated with higher caspase expression shortly before and after pupation [9]. Among the different protective measures triggered by the insect immune system to thwart pathogen infection, host cell suicide through apoptosis can significantly reduce viral replication, dissemination and infectivity [10].

A major family of evolutionarily conserved cysteine-dependent aspartate-specific proteases, called caspases, plays a central role in apoptosis. Studies on alpha-proteobacteria, closely related species of the free-living ancestor of mitochondria, revealed the presence of genes similar to caspases, suggesting that ancestor of eukaryotic caspases derived from mitochondrial endosymbionts [11]. Ancestors of caspase genes then evolved into 'metacaspases' in plants and fungi, 'paracaspases' in slime, molds and animals and in 'true caspases' in animals. The number of "true" caspase genes differs greatly among animal lineages with as little as 4 genes in *Caenorhabditis elegans *[12], 5 in the sea anemone *Nematostella vectensis*, 7 in the fruit fly *Drosophila melanogaster *[13], 10 and 11 in the mosquitoes *Anopheles gambiae *and *Aedes aegypti *respectively [14], 12 in human [15,16], to up to 31 in the sea urchin *Strongylocentrotus purpuratus *[17]. It has been hypothesized that the host-pathogen interaction has been one of the major evolutionary forces shaping the apoptotic machinery and therefore the caspase repertoire [17,18].

The caspase family is divided into two groups: the inflammatory caspases and the apoptotic caspases composed of the initiator and the effector subgroups. To date, dedicated inflammatory caspases seem to be restricted to mammals. Although a non-apoptotic role of some lepidopteran caspases should not be excluded at this stage, the description of inflammatory caspases will not be considered further here. Apoptotic caspases are synthesized in the cell as catalytically inactive forms. All members of the caspase family share a canonical structure consisting of a prodomain and a catalytic domain named "peptidase C14" (Caspase domain pfam PF00656). The catalytic domain is formed by 2 subunits, a large one of about 20 kDa, also known as p20, and a small one of about 10 kDa, also referred as p10. Upon proteolytic processing of the proenzyme, the two subunits form a heterodimer and the active caspase enzyme is made of the association of two of these heterodimers. The enzymatic activity of caspases shows strong cleavage site specificity towards a four amino-acid motif ending with an aspartate residue [19-21]. In contrast to the catalytic domain, which is structurally conserved, the prodomain varies significantly among caspases. The characteristic binding sites (^L^/_S_^T^/_S_HG) and active sites (Q^A^/_R_C^R^/_Q_G) are conserved in all caspases except in *D. melanogaster *Dronc [22]. Whereas effector caspases have a short prodomain of about 30 amino acids, initiator caspases have a prodomain of 80 or more amino acids [23]. Long prodomains usually harbor structures belonging to the Death Domain superfamily, such as Death Effector Domain (DED) or Caspase Recruitment Domain (CARD), which are involved in the recruitment and subsequent activation of initiator caspases by death receptors or by the apoptosome [24].

**Figure 1:**
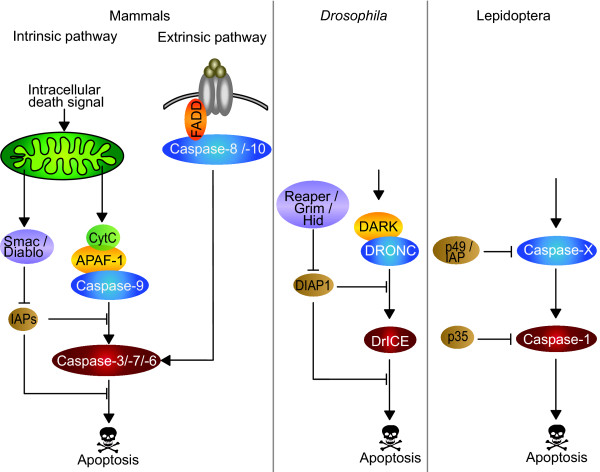
**Apoptotic pathway in mammals, *Drosophila *and Lepidoptera**. Homologs of caspases and caspase regulators across species are indicated by the same color. Initiator and effector caspases are colored in blue and red respectively. The death receptor is colored in grey, the adaptor protein in orange, the protein forming the apoptosome in yellow, the apoptotic inducers in purple, and the caspase inhibitors in brown.

In the pro-apoptotic proteolytic cascade, initiator caspases are activated first, and these in turn activate the effector caspases. The latter are responsible for the cleavage of many cellular components [25], leading to the characteristic features of apoptotic cells such as DNA fragmentation and membrane blebbing [26]. In mammals, in the extrinsic pathway, the binding of a "death" ligand to its receptor changes the receptor conformation, allowing the recruitment of an adaptor protein such as FADD and of initiator Caspase-8. The formation of this complex leads to the autoactivation of Caspase-8 (Figure 1). The intrinsic pathway is activated in response to intracellular signals which induce a permeabilization of the mitochondria outer membrane, allowing the release in the cytosol of several proteins such as Smac/Diablo and Cytochrome c. Cytochrome c forms a protein complex with Apaf 1 called the apoptosome, which recruits the initiator Caspase-9 leading to its subsequent activation. Caspase-8 and -9 are both able to activate the effector caspase-3, -6 and -7 (Figure 1). In *Drosophila*, there is no evidence of an extrinsic pathway, and the formation of the apoptosome with Dark, the homolog of Apaf 1, and Dronc, the homolog of Caspase-9, does not require Cytochrome c [27]. Dronc then activates the effector caspase DrICE. Homologs of the core components of the apoptosis machinery found in *Drosophila *have also been described in mosquitoes, suggesting that apoptosis is regulated in a similar fashion in mosquitoes [14].

In Lepidoptera, very little is known about apoptotic molecular pathways (Figure 1). The discovery in the early 1990's of p35, a baculovirus pan-caspase inhibitor [28], has led to the characterization of the first lepidopteran caspase, Sf-caspase-1, from the Sf9 cell line derived from the noctuid moth *Spodoptera frugiperda *[29]. Later, the likely orthologs from *S. littoralis *[30], *Helicoverpa armigera *[31] and *Trichoplusia ni *[32] were also characterized. Members of this caspase-1 group have been shown to act as effector caspases, but so far no other classes of caspases have been described in Lepidoptera. The existence of an "apical" caspase responsible for the activation of Sf-caspase-1 has been suggested [33]. Furthermore, 2 other baculovirus proteins, p49 and IAP [34,35] have been shown to inhibit apoptosis in Sf9 cells by blocking the processing of Sf-caspase-1, suggesting that the likely target of these 2 inhibitors may be an apical caspase, tentatively named Sf-caspase X. So far, this caspase X has not been characterized, but these results suggest that, in Lepidoptera, apoptosis requires a cascade of caspase activation, similar to other organisms.

In an attempt to characterize the caspase gene family in lepidopteran insects, we first surveyed the recently sequenced genome of the silkworm *Bombyx mori *[36], as well as publicly available and 'in-house' EST datasets. From these, we retrieved 66 transcripts encoding putative caspases from 27 species of butterflies and moths. Phylogenetic analyses showed that these genes clustered into 6 distinct clades (Caspase-1 to -6), three of which are classified as putative effector caspases, two corresponding to putative initiator caspases and one that could not be readily classified as either. We propose a naming convention for these genes based on our phylogenetic analyses. Finally, we discovered that one of the caspase subfamilies (caspase-2) is absent from the genome of the silkworm, has evolved from a tandem gene duplication of caspase-1, is under purifying selection which however is more relaxed than for caspase-1, and is likely to be restricted to species of the family Noctuidae.

## Results and discussion

### Lepidopteran caspases cluster into six distinct clades

**Figure 2:**
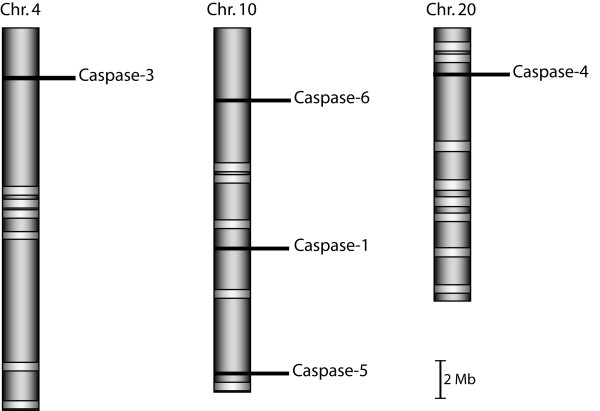
**Schematic representation of the chromosomal localization of caspase genes on the Silkworm genome**. The keyword "peptidase C14" was used to perform a search on the Kaikobase keyword search engine (http://sgp.dna.affrc.go.jp/KAIKObase/). Putative gene sequences were retrieved and checked for conserved caspase domains.

**Figure 3:**
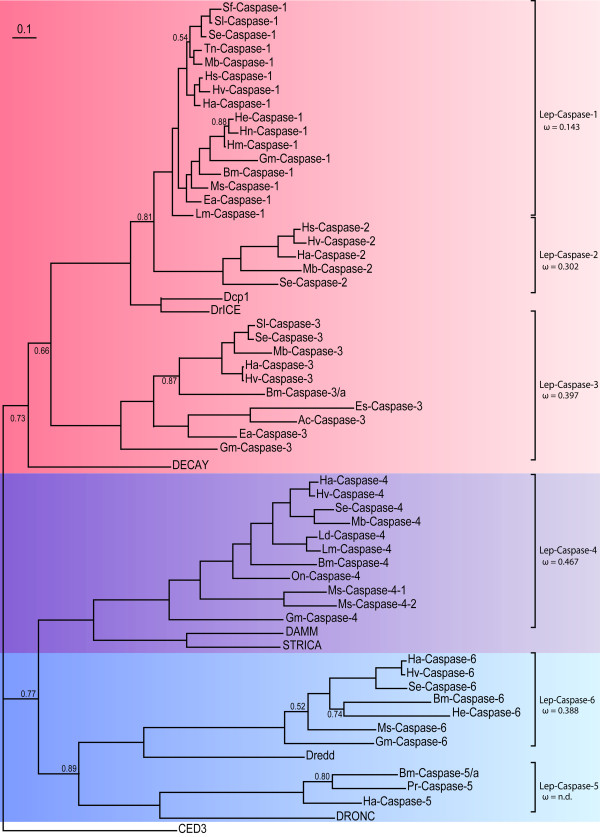
**Phylogenetic relationships between caspase sequences found in Lepidoptera in relation with their *Drosophila *counterparts**. Amino acid alignments of caspase sequences without their prodomain were used to build a phylogenetic tree using a Bayesian inferred method with the *C. elegans *caspase Ced3 as an outgroup. Lepidopteran caspase clades were determined by branching patterns and are represented by brackets on the right. Posterior probabilities are shown only for nodes below 0.9. dN/dS ratio (ω) for each clade is mentioned below the clade name, except for Lep-Caspase-5 for which the number of sequences was too low to determine it.

As the genome of the silkworm *Bombyx mori *is the sole fully sequenced genome for insects of the order Lepidoptera to date, we first searched it for the presence of putative caspase genes (Figure 2). We identified a total of 5 genes, one on chromosome 4 (Caspase-3), three at various places on chromosome 10 (Caspase-1, -5 and -6) and one on chromosome 20 (Caspase-4) (Figure 2). Our findings are in agreement with a genome-wide analysis of the *Bombyx mori *genome for apoptosis-related genes, in which these 5 caspase genes have also been identified [37]. To get further insight into this gene family, we then mined publicly available as well as "in-house" EST datasets for transcripts encoding putative caspase enzymes. We retrieved a total of 66 transcripts corresponding to 63 different genes, from 27 species of moths and butterflies spanning 11 lepidopteran families (Additional file 1). Caspase sequences were accepted if they fulfilled the following criteria: presence of the characteristic binding site (^L^/_S_^T^/_S _H G) and/or active site (QAC^R^/_Q_G), and level of amino acid identity (for complete amino acid alignments, see Additional files 2, 3, 4, 5, 6 and 7). A Bayesian inferred phylogenetic analysis clustered these sequences into six distinct clades (Figure 3), revealing an extra caspase gene family that was not found in the *B. mori *genome (Caspase-2). This observed number of caspase genes in Lepidoptera is similar to the seven genes found in *Drosophila *species: Drice, Dcp-1, Decay, Damm, Dronc, Dredd and Strica (also known as Dream) [13].

To facilitate our analysis at this stage, we decided to establish a naming convention for these genes based on the results of the phylogenetic analyses. The first caspase fully characterized for a lepidopteran insect has been named Caspase-1 preceded by the initials Sf, corresponding to the name of the species *Spodoptera frugiperda *[29]. We extended this convention by naming each caspase starting with the initials of the species from which it has been isolated, followed by "-Caspase" and by a number depending on which clade the sequence clustered into. Clades were arbitrarily numbered Caspase-1 to Caspase-6. The term "Lep" was added to the cluster name to differentiate lepidopteran caspases from sequences derived from other organisms. When splicing variants were found (see below), we used the nomenclature proposed by Alnemri *et al *in which each variant is identified by a letter [38]. Here we stress the point that our nomenclature does not reflect any orthology between lepidopteran caspases and mammalian ones. For example, Lep-Caspase-2 is not an ortholog of Human caspase-2.

### Classification of the lepidopteran caspases

**Figure 4:**
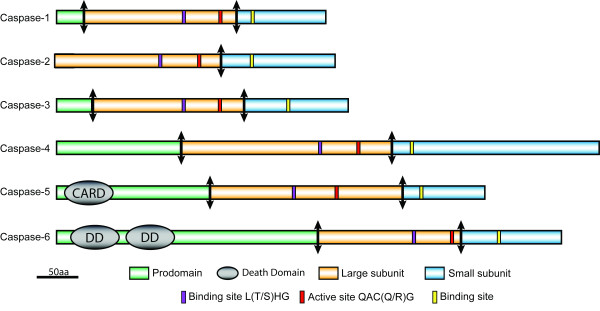
**Predicted domain structure of lepidopteran caspases**. Conserved domains were predicted using ExPASy ScanProsite. Active and binding sites are indicated. Arrows represent the putative cleavage sites between the prodomain and the large subunit, and between the large and small subunits. Note that no suitable cleavage site between prodomain and large subunit could be found in Lep-Caspase-2 sequences.

The phylogenetic analysis clustered Lep-Caspase-1, -2 and -3 with Dcp-1, Drice and Decay (Figure 3). These 3 *Drosophila *caspases have been classified as effectors due to their short prodomain, their substrate specificity toward the tetrapeptide DEVD and their ability to cleave the Poly (ADP-ribose) polymerase and/or p35 [39-42]. Lep-Caspase-1 and -3 also harbor a short prodomain, characteristic of effector caspases (Figure 4). However, no suitable cleavage site between the prodomain and the large subunit could be found in Lep-Caspase-2, and an amino acid alignment of Lep-Caspase-1 and -2 sequences suggests that the latter lacks a prodomain (Additional file 8). Caspase-1 has already been characterized in *S. frugiperda *and *S. littoralis*. These studies demonstrated substrate specificity toward DEVD [30] and the ability to cleave p35 similar to the *Drosophila *effector caspases [29]. Although Drice and Dcp-1 are highly similar in sequence, several studies have shown that Drice and not Dcp-1 is the main effector caspase in *Drosophila *[41,43]. A study comparing the effect of single and double mutants for Drice and Dcp-1 showed that these two caspases have an overlapping function [44]. The role of Decay has still to be clarified. Finally, based on another phylogenetic analysis including all the known mosquito caspase sequences [45], we find no clear orthology between lepidopteran and dipteran effector caspases, which is due to the rapid evolution and diversity of this gene family (Additional file 9).

Like the *Drosophila *Dronc, to which it is closely related, and human Caspase-9, Lep-Caspase-5 harbors a CARD domain within its long prodomain (Figure 4). Typical CARD domains are constituted by a bundle of 6 alpha helices. An amino acid alignment of the prodomain of CARD containing caspases, including *B. mori *caspase-5, shows a low amino acid conservation, but a high conservation of the position of the 6 alpha helices (additional file 10). CARD domains are involved in the interaction of initiator caspases with Ark/Apaf1, to form the apoptosome which is required for the activation of Dronc/human Caspase-9 [46,47]. It has been shown that *dronc *is ubiquitously expressed during the development of *Drosophila *and that its expression is stimulated by ecdysone during metamorphosis [22]. Lep-Caspase-6 is closely related to *Drosophila *Dredd. The presence, in the long prodomain, of 2 motifs composed of 6 alpha helices, forming a three-dimensional structure characteristic of the Death Domain family (Figure 4), strongly supports its classification as an initiator caspase [24]. An amino acid alignment of *B. mori *caspase-6 with other Death Domain-containing caspases from human and Diptera shows again a low amino acid conservation, but the position of the alpha helices is conserved (Additional file 11). Despite the first description of Dredd as a potential initiator caspase [48], it now appears to be more important in activating the innate immune response upon infection by Gram negative bacteria [49]. Based on a phylogenetic analysis including all the known mosquito caspase sequences, it is clear that Lep-Caspase-5 and Lep-Caspase-6 are the orthologs of dipteran Dronc and Dredd, respectively (Additional file 9).

Despite a relatively long prodomain, Lep-Caspase-4 does not harbor any known Death Domain which would have supported its classification as an initiator caspase (Figure 4). The phylogenetic analyses show a relationship with the *Drosophila *caspases Damm and Strica, for which the function has not been clearly defined yet. Lepidopteran Caspase-4 sequences share about 20% amino acid identity with Strica/Damm homologs from flies and mosquitoes, but do not harbor any serine-threonine-rich regions in the prodomain [50]. Although Damm and Strica are very similar in sequence, they appear to play different roles in *D. melanogaster*: Damm seems to be an effector caspase [51], whereas Strica seems to be an initiator caspase, exhibiting redundant activity with Dronc during oogenesis based on genetic studies [52]. Within the 12 sequenced *Drosophila *species, Damm is a more recent duplication of the ancestral Strica, as Damm is restricted to the melanogaster and obscura groups [45]. Perhaps the ancestral Strica was an initiator caspase in Diptera. Another unusual feature of Lep-Caspase-4 is its unique small subunit. It is twice as long as any other small subunit described so far, which usually ends 60 to 80 amino acids after the conserved serine residue (Ser^428^) involved in substrate binding (Additional files 2, 3, 4, 5, 6 and 7). In addition, alignments (Additional file 5) show that the sequence corresponding to the last 140 residues of the small subunit is more variable compared to the rest of the subunit. However some residues are conserved in all sequences, including 3 cysteine residues potentially involved in the formation of secondary structures. These features indicate that Lep-Caspase-4 is a peculiar caspase, the function of which cannot be solely assessed by sequence comparisons.

### Alternative splicing of Caspase-3 and -5 in *B. mori*

In *B. mori*, two isoforms of Caspase-5 have been identified, a long form (-5/a) and a short form (-5/b) in which the exon encoding the sequence corresponding to the catalytic site is spliced out (See Additional file 12 for the intron-exon structure of lepidopteran caspases). The short form still possesses an intact prodomain and small subunit. In human, Caspase-9 splicing variants have been identified. In one of them, Caspase-9S, the catalytic site is missing and this isoform has been shown to be a dominant-negative inhibitor of apoptosis, by blocking the Caspase-9/Apaf1 interaction [53]. Enzymatically inactive caspase homologs that have arisen by gene duplication events have been recently described in Diptera [45]. These caspase-like decoy molecules may have acquired the ability to regulate other caspases. The short form of Bm-caspase-5 might have evolved a similar decoy function by alternate splicing, however we could not find any separate genes encoding caspase-like decoy molecules in our analyses. Although several isoforms of Caspase-5 were observed in *B. mori*, only a single transcript could be detected in *H. armigera*. In addition, the sequences corresponding to exon 6 and 7 in *B. mori *are part of a single exon in *H. armigera*, so a product like *B. mori *Caspase-5b could not be produced by alternate splicing in the latter species.

Three splicing variants were detected for Bm-Caspase-3 (Additional files 4 and 12), all of them harboring both catalytic and binding sites. Alternative splicing was not observed for Ha-Caspase-3. It has been suggested that catalytically active isoforms may differ in specificity and efficiency, which could fine-tune caspase activity through hetero-dimerization, resulting in amplification or inhibition of apoptosis [54].

### A noctuid-specific caspase gene arose from duplication of caspase-1

**Figure 5:**
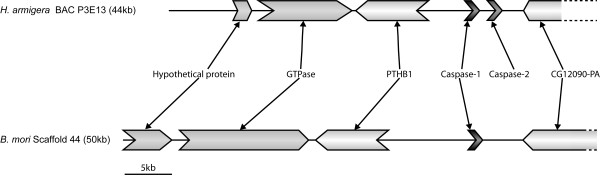
**Representation of the genomic region surrounding Caspase-1 in *B. mori *and *H. armigera***. Deduced from a *B. mori *scaffold and the sequencing of a *H. armigera *BAC clone. The Caspase-1 and Caspase-2 genes are found in tandem on the *H. armigera *genome. Gene annotation was performed using Kaikogaas.

Thorough mining of lepidopteran EST datasets for putative Caspase-2 led us to find the corresponding genes only in species of the family Noctuidae (Figure 3 and Additional file 1). No ortholog of Caspase-2 could be found in the silkworm genome (Figure 2). Taking into account that the amino acid identity between Lep-Caspase-1 and Lep-Caspase-2 sequences is relatively high (ranging between 47 and 53%), we hypothesized that both genes may have arisen, in Noctuidae, from a tandem gene duplication event. To test this hypothesis, we screened a *H. armigera *BAC library using probes designed according to either the Ha-Caspase-1 or the Ha-Caspase-2 sequence. A BAC clone (clone P3E13, Genbank accession HQ645847) hit by both probes was identified and selected for sequencing. The Caspase-1 and -2 genes were found, in a 43 kb fragment of this BAC, to be organized in tandem, separated by only 3 kb of non-coding DNA, were in the same orientation (Figure 5) and had no intron (Additional file 12), confirming our hypothesis. We then compared the organization of the genes surrounding Caspase-1 between *H. armigera *and *B. mori *(Figure 5), we found a high degree of micro-synteny in this region of the genome between the two species, similar to what has been reported in recent studies [55,56].

To examine the relative rates of sequence evolution within the Caspase-1 and -2 subfamilies since divergence, pairwise comparisons of sequences obtained from noctuid species were performed by applying the Nei-Gojobori algorithm [57] (see Additional file 13 for the P-distances of synonymous and non-synonymous substitutions). Mean of synonymous substitutions per site (d_S_) was 0.480 and 0.549 within Caspase-1 and Caspase-2 respectively; mean d_S _between Caspase-1 and -2 is 0.646, indicating saturation. Mean non-synonymous substitutions per site (d_N_) was 0.047 for Caspase-1 and 0.170 for Caspase-2. We used the Codon-based Z-test [58] to test the null hypothesis d_S _= d_N _which would indicate that both genes are neutrally evolving. We could reject this null hypothesis as we found that d_N _is significantly smaller than d_S _(Caspase-1: Z = -17.000, p < 0.001; Caspase-2: Z = -14.138, p < 0.001), indicating a strong purifying selection on both genes. We also tested whether synonymous and non-synonymous substitution rates differ between the two genes (d_S*Casp1 *_= d_S*Casp2*_and d_N*Casp1 *_= d_N*Casp2*_, same Z-test as before) we could not reject that d_S*Casp1 *_= d_S*Casp2*_(Z = -2.03, p > 0.05), but the non-synonymous substitution rate in Caspase-2 was significantly higher compared to the one in Caspase-1 (Z = -8.853, p < 0.001). The coefficient of functional divergence [59], which measures the overall difference between site-specific substitutions among the two subfamilies is θ = 0.57, which is significantly greater than zero (likelihood ratio test, χ^2 ^= 7.31, P < 0.01). These results strongly suggest that Caspase-2, though also under purifying selection following the duplication event, has evolved under much more relaxed selective constraints than Caspase-1. It has been well documented that gene duplication is often followed by a period of relaxed selective constraints on one of the duplicate, allowing it to accumulate more mutations [60,61]. Although it has been suggested that gene duplication occurs at a high rate of 0.01/gene/million years, the most common fate of duplicated genes is silencing and loss [62]. In some cases however, the duplicate is retained as functional gene and either a new function is acquired by one of the copies or the ancestral function is maintained or subdivided between the two copies [63]. The fact that the cleavage site differ between the two subunits (TETD for Caspase-1 and xETD for Caspase-2), and that Caspase-2 may lack a prodomain, suggests that they might be activated by different signals. This could indicate a subfunctionalization of Caspase-2 in Noctuidae.

### Ms-Caspase-4 subfamily has evolved through duplication events

**Figure 6:**
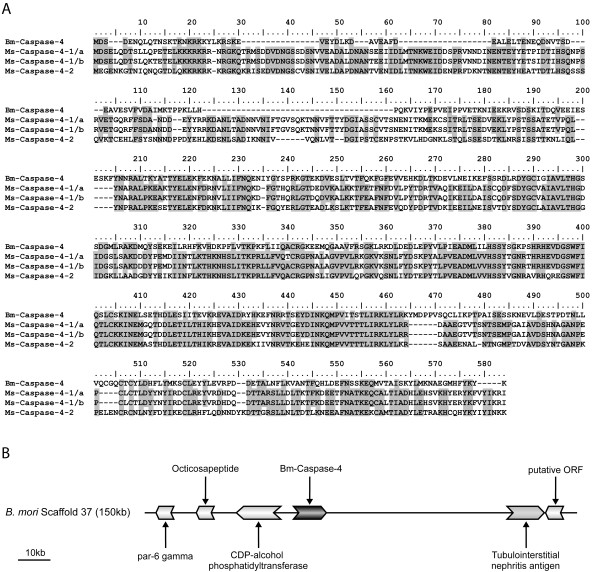
**Recent gene duplications of the Caspase-4 gene in *M. sexta***. (A) Amino acid alignment of the Caspase-4 sequences from *B. mori *and *M. sexta*. Identical amino acids are shaded in grey. (B) Schematic representation of the genomic region surrounding the Caspase-4 gene deduced from a *B. mori *scaffold. Gene annotation was performed using Kaikogaas.

Several contigs corresponding to putative Caspase-4 transcripts were identified from an EST dataset derived from a *M. sexta *larval midgut cDNA library, sequenced by 454 pyrosequencing [64]. Analyses of complete sequences obtained by RACE-PCR resulted in 3 distinct cDNAs. Two of them, Ms-Caspase-4-1/a and -4-1/b, differ only by 20 single nucleotide polymorphisms (SNPs), 8 of which are non-synonymous, as well as an extra codon encoding ASN^113 ^in Ms-Caspase-4-1/b (Figure 6A). The third sequence, Ms-Caspase-4-2, encodes a protein sharing ~63% amino acid identity with both Ms-Caspase-4-1/a and -4-1/b (Figure 6A). The occurrence of several unique transcripts for Lep-Caspase-4 has only been found in that species so far. A closer look at the region where Bm-Caspase-4 is located on the silkworm genome did not reveal any extra Lep-caspase-4 genes or even pseudogenes nearby Bm-Caspase-4 (Figure 6B). These results suggest that Caspase-4 has undergone at least two duplication events in *M. sexta*. A phylogenetic study of dipteran caspases suggest that Damm and Strica, the homologs of Lep-Caspase-4, have arisen by duplication in the *melanogaster *and *obscura *clades, while only Strica is present in the other drosophilid clades [45]. In mosquitoes, the ancestor of Damm/Strica has undergone many duplication events after the divergence of the *Anopheles gambiae, Culex quinquefasciatus *and *Aedes aegypti *lineages. Two homologs of Damm/Strica have been described in *A. gambiae*, as well as 2 in *C. quinquefasciatus *and 4 in *A. aegypti *[14,45]. All of the duplicates are potentially active since they all harbor the critical amino acids involved in binding and active sites. However, no biochemical characterization has been carried out on these caspases so far. The paucity of EST data for members of the family Sphingidae does not allow us to conclude whether the duplication of Lep-Caspase-4 is specific only to *M. sexta *or if it has also happened in sister species.

## Conclusions

Despite obvious limitations of EST datasets in term of available species and transcriptome coverage, we were able to identify 63 caspase genes coming from 27 different lepidopteran species. Based on the phylogenetic analyses, we showed that the lepidopteran caspase family is represented by at least 5 members. Nevertheless, the biochemical characterization of these caspases has to be performed to clarify the exact function and their potential interactions. The main shaping forces of the caspase gene family in mammals as well as in other insects, such as mosquitoes, are gene deletions and gene innovations by duplication events [16,45]. Although we cannot assess whether deletions of caspase genes has happened in Lepidoptera, our data suggest that gene duplication is one of the shaping forces of this gene family in these insects. Availability in the near future of whole genome sequences and transcriptomes from other species of the order Lepidoptera as well as from other insect orders, will improve our understanding of the complexity of this gene family and its evolutionary history in insects in general.

## Methods

### Preparation of cDNA libraries and EST sequencing

Generation of cDNA libraries for *T. ni *whole larvae [65]; *Plutella xylostella, Pieris rapae, Colias eurytheme *and *Anthocharis cardamines *whole larvae [66]; and for *H. armigera *midgut and *S. littoralis *whole larvae [55] were previously described. TRIzol Reagent (Invitrogen) was used to isolate total RNA from whole larvae or dissected larval tissues of *Pontia daplidice, Eucheira socialis, Lymantria dispar, Lymantria monacha, G. mellonella, H. virescens, Mamestra brassicae*, and *Spodoptera exigua*. Normalized, full-length, enriched cDNA libraries were generated using both the Creator SMART cDNA library construction kit (BD Clontech) and the Trimmer Direct cDNA normalization kit (Evrogen), generally following the manufacturer's protocol but with several important modifications. In brief, 2 μg of poly(A)+ mRNA was used for each cDNA library generated. Reverse transcription was performed with a mix of several reverse transcriptases for 1 h at 42°C and 90 minutes at 50°C. cDNA size fractionation was performed with SizeSep 400 spun columns (GE Healthcare) that resulted in a cutoff at ~200 bp. The full-length, enriched cDNAs were cut with SfiI and ligated to the SfiI-digested pDNR-Lib plasmid vector (Clontech). Ligations were transformed into E. coli ELECTROMAX DH5α-E electro-competent cells (Invitrogen). Plasmids from bacterial colonies grown in 96 deep-well plates were prepared using the Robot 96 Plasmid isolation kit (Eppendorf) on a Tecan Evo Freedom 150 robotic platform (Tecan). Single-pass sequencing of the 5' termini of cDNA libraries was carried out on an ABI 3730 xl Automatic DNA Sequencer (PE Applied Biosystems). Vector clipping, quality trimming, and sequence assembly were done with the Lasergene 8 software package (DNAStar Inc.). BLAST searches were conducted on a local server using the National Center for Biotechnology Information (NCBI) blastall program. Sequences were aligned using ClustalW software [67].

### Database mining for putative caspase sequences

The protein sequence of Sf-Caspase-1 (AF548387) [29] was used as a query to perform TBLASTN searches of lepidopteran EST databases publicly available on NCBI dbEST, on InsectaCentral [68,69], or in-house (Additional file 1). Sequences were accepted as being "caspases" when presenting the characteristic binding site (^L^/_S _^T^/_S _H G) and/or active site (QAC^R^/_Q_G). Some exceptions were made when partial amino acid sequences presented a strong amino acid identity with sequences previously accepted as caspases.

### Caspase genes amplification and sequencing

Specific primers (Additional file 14) were designed in order to re-amplify BmCasp-4, and -5, as well as HaCasp-1, -2, -3 and -4 using total RNA prepared respectively from *B. mori *and *H. armigera *whole larvae as starting material. In order to get full length cDNA, 5'-and 3'-RACE were performed for HaCasp-5, and -6, BmCasp-4, MsCasp-1, -4-2 and -6 as well as EaCasp-1 and -3 using the SMART^® ^RACE cDNA amplification kit (Clontech), following the manufacturer's instructions. Total RNA from *H. virescens, H. subflexa *and *H. armigera *larvae was extracted using TRIzol, according to the manufacturer's specifications. Forward and reverse specific primers designed according to the HvCasp-6 sequence (Additional file 14) were used to amplify HaCasp-6 from *H. armigera *cDNAs. Forward and reverse specific primers designed according to the HaCasp-1 and -2 sequences (Additional file 14) were used to amplify Casp-1 and -2 from *H. virescens *and *H. subflexa *cDNAs. Finally, a *H. armigera *draft genome assembly generated by 454 pyrosequencing (unpublished data) was searched using BmCasp-5 as query to identify a homolog in *H. armigera*. Forward and reverse primers (Additional file 14) were designed based on the sequences of contigs 28382 and 105066 in order to amplify HaCasp-5 sequence from cDNAs extracted from *H. armigera *HaAM1 cells [70] treated with 75 μM ecdysone. All amplicons were then ligated in pCR2.1-Topo^® ^vector (Invitrogen). Ligations were transformed into *E. coli *TOP10 chemically competent cells (Invitrogen) and plasmid mini-preparation and sequencing were performed as mentioned above.

### Sequence alignment and phylogenetic analysis

Deduced amino acid sequences from caspase transcripts retrieved from the various databases searched were aligned together with the *Drosophila *caspases (Drice, Dcp-1, Decay, Dronc, Dredd, Damm and Strica) and with the *C. elegans *caspase Ced3 as an outgroup, using the FFT-Ns-i strategy implemented in the MAFFT alignment program [71]. After removing the prodomain, the amino acid alignment was then used for the phylogenetic analyses. Only sequences containing the complete open reading frame (ORF) or partial sequences containing at least the part encoding the complete large and small subunits were used to build the phylogenies. The phylogenetic reconstruction was done by Bayesian inference using MrBayes 3.1 [72]. The prior was set for the amino acid model to mix, thereby allowing model jumping between fixed-rate amino acid models. Markov Chain Monte Carlo runs were carried out for 10,000,000 generations after which log likelihood values showed that equilibrium had been reached after the first 400 generations in all cases, and those data were discarded from each run and considered as 'burnin'. Two runs were conducted for the dataset showing agreement in topology and likelihood. To determine which clusters partial caspase sequences belong to, amino acid sequences were aligned with MAFFT using the FFT-Ns-i strategy; and a phylogenetic tree was generated by Neighbor joining (1000 replicates) using MEGA4 software [73]. Relative-rate tests were applied to compare rates of evolution in the Caspase-1 and Caspase-2 subfamilies. Synonymous and non synonymous substitution rates were determined by the Nei and Gojobori algorithms [57] as implemented in MEGA4. Statistics were performed using the Codon-based Z-test, which corresponds to a t-test with an infinite degree of freedom [74]. The coefficient of functional divergence θ was calculated using the program DIVERGE 2.0 [75]. This coefficient ranges between 0 and 1 and measures the overall degree to which site-specific substitution rates differ; values significantly greater than zero indicate rate differences among homologous sites in the two subfamilies. In order to characterize the primary structure and domain architecture of the different caspases, their predicted amino acid sequences were submitted to ExPASy ScanProsite [76]. For comparison to dipteran caspases, sequences from *D. melanogaster, Culex quinquefasciatus, Anopheles gambiae*, and *Aedes aegypti *were extracted from Supplementary Figure S1 of ref [45].

### BAC library screening and sequencing

Nylon filters from a *Helicoverpa armigera *BAC library established from the strain Toowoomba were washed, blocked, and hybridized with horseradish peroxidase-labelled DNA fragment containing part of the *H. armigera *Casp-1 and Casp-2 genes. Labeling, hybridization, and probe detection were done according to specifications in the ECL DNA labeling and detection kit (GE Healthcare). Positive clones were isolated from glycerol stocks, grown in Terrific Broth, and BAC DNA was isolated with the Nucleobond Xtra Maxi Kit according to the manufacturer's instructions (Macherey-Nagel). BAC genomic DNA quantities were estimated spectrophotometrically on a Nanodrop ND 1000 (Peqlab biotechnologie GmbH). Positive clones were digested by EcoR1 and HindIII, blotted and re-hybridized first with Ha-Caspase-1 probe then with Ha-Caspase-2 to identify positives clones for the 2 probes. BAC DNA was sheared into two different size ranges (1.5 -2 kb and 4-5 kb) with a hydroshear device (Molecular devices), blunted with the Quick blunting kit (New England Biolabs), isolated from agarose gel, column purified, and ligated into the pUC19-SmaI vector (Fermentas). Ligations were transformed into *E. coli *ELECTROMAX DH5α-E electrocompetent cells (Invitrogen). Plasmid preparation, sequencing, and assembly were performed as mentioned above.

### Intron-Exon structure

*Bombyx mori *caspase cDNA sequences were compared to the genomic sequences retrieved from KAIKObase [77,78] (Caspase-1: BGIBMGA006940, Caspase-3: BGIBMGA006131, Caspase-4: BGIBMGA004420, Caspase-5: BGIBMGA002841, Caspase-6: BGIBMGA006726) using the Spidey software available at NCBI. The same comparison was done using *H. armigera *cDNA sequences and the genomic sequences retrieved from the *H. armigera *draft genome assembly mentioned above.

## Competing interests

The authors declare that they have no competing interests.

## Authors' contributions

JC, YP and DGH conceived the study. JC, YP and HV performed experiments. JC, YP and HV analyzed the data. JC and YP wrote the initial manuscript and all authors contributed to the preparation of the final version. All authors read and approved the final manuscript.

## Supplementary Material

Additional file 1 **Table S1**. Details of the caspase sequences characterized in this study. Click here for file

Additional file 2 **Figure S1**. Amino acid alignment of Lep-Caspase-1 sequences. Click here for file

Additional file 3 **Figure S2**. Amino acid alignment of Lep-Caspase-2 sequences. Click here for file

Additional file 4 **Figure S3**. Amino acid alignment of Lep-Caspase-3 sequences. Click here for file

Additional file 5 **Figure S4**. Amino acid alignment of Lep-Caspase-4 sequences. Click here for file

Additional file 6 **Figure S5**. Amino acid alignment of Lep-Caspase-5 sequences. Click here for file

Additional file 7 **Figure S6**. Amino acid alignment of Lep-Caspase-6 sequences. Click here for file

Additional file 8 **Figure S7**. Amino acid alignment of noctuid-derived caspase-1 and caspase-2 sequences. Click here for file

Additional file 9 **Figure S8**. Phylogenetic relationship of caspases sequences found in Lepidoptera and Diptera. Click here for file

Additional file 10 **Figure S9**. Alignment of Bm-Caspase-5 with the prodomains of Drosophila Dronc, Aedes aegypti Ae-Dronc and human Caspase-1, -2 and -9. Click here for file

Additional file 11 **Figure S10**. Alignment of Bm-Caspase-6 with the prodomains of Drosophila Dredd, Aedes aegypti Ae-Dredd and human Caspase-8 and -10. Click here for file

Additional file 12 **Figure S11**. Intron-exon structure of lepidopteran caspases. Click here for file

Additional file 13 **Figure S12**. P-distances of synonymous and non-synonymous substitutions among the Noctuids caspase-1 and -2. Click here for file

Additional file 14 **Table S2**. Primer sequences used for amplification of caspase transcripts. Click here for file
