# Supplementary material for: A comprehensive characterization of the caspase gene family in insects from the order Lepidoptera
Source: BMC Genomics. 2011 Jul 8;12:357. doi: 10.1186/1471-2164-12-357 (PMC3141678; doi:10.1186/1471-2164-12-357)
Supplement: Additional file 1 — Table S1. Details of the caspase sequences characterized in this study. [file 1471-2164-12-357-S1.PDF]

**Table S1.** Details of the caspase sequences characterized in this study. Sequences marked with an asterisk are new to this study.

| Species | Common name | Family | Gene | Database | accession |
|---|---|---|---|---|---|
| **Caspase 1** | | | | | |
| *Antheraea assama* | Muga silkworm | Saturniidae | AaCASP-1 | Genbank | FG217522 |
| *Bicyclus anynana* | Squinting bush | Nymphalidae | BaCASP-1 | Genbank | GE695124 |
| *Bombyx mori* | Silkworm | Bombycidae | BmCASP-1 | Genbank | AF448494 |
| *Epiphyas postvittana* | light brown apple | Tortricidae | EpCASP-1 | Genbank | EV811051 |
| *Euphydryas aurinia* | Marsh fritillary | Nymphalidae | EaCASP-1 | Genbank | HM234680* |
| *Galleria mellonella* | Greater Wax Moth | Pyralidae | GmCASP-1 | in house | HQ328948* |
| *Heliconius erato* | Red Postman | Nymphalidae | HeCASP-1 | Genbank | EF207976 |
| *Heliconius numata* | Numata longwing | Nymphalidae | Hn-CASP-1 | Insecta central | IC33419AbAorf6448 |
| *Heliconius melpomene* | | Nymphalidae | HmCASP-1 | Genbank | EF211965 |
| *Helicoverpa armigera* | Cotton Bollworm | Noctuidae | HaCASP-1 | Genbank | EF688063 |
| *Heliothis subflexa* | | Noctuidae | HsCASP-1 | PCR | HQ328949* |
| *Heliothis virescens* | Tobacco Budworm | Noctuidae | HvCASP-1 | PCR | HQ328950* |
| *Lymantria monacha* | Black Arches | Lymantriidae | LmCASP-1 | in house | HQ328951* |
| *Manduca sexta* | Tobacco hornworm | Sphingidae | MsCASP-1 | Genbank | HM234675* |
| *Mamestra brassicae* | Cabbage Moth | Noctuidae | MbCASP-1 | in house | HQ328952* |
| *Spodoptera exigua* | Beet Armyworm | Noctuidae | SeCASP-1 | in house | HQ328953* |
| *Spodoptera frugiperda* | Fall Armyworm | Noctuidae | SfCASP-1 | Genbank | U81510 |
| *Spodoptera littoralis* | African Cotton | Noctuidae | SlCASP-1 | Genbank | AF548387 |
| *Trichoplusia ni* | Cabbage Looper | Noctuidae | TnCASP-1 | Genbank | AY159381 |
| **Caspase 2** | | | | | |
| *Helicoverpa armigera* | Cotton Bollworm | Noctuidae | HaCASP-2 | in house | HQ328954* |
| *Heliothis subflexa* | | Noctuidae | HsCASP-2 | PCR | HQ328955* |
| *Heliothis virescens* | Tobacco Budworm | Noctuidae | HvCASP-2 | PCR | HQ328956* |
| *Mamestra brassicae* | Cabbage Moth | Noctuidae | MbCASP-2 | in house | HQ328957* |
| *Spodoptera exigua* | Beet Armyworm | Noctuidae | SeCASP-2 | in house | HQ328958* |
| **Caspase 3** | | | | | |
| *Anthocharis cardamines* | Orange Tip | Pieridae | AcCASP-3 | in house | HQ328959* |
| *Bombyx mori* | Silkworm | Bombycidae | BmCASP-3 | Genbank | DQ360829 (variant a)<br>AY885228 (variant b)<br>DQ360830 (variant c) |
| *Eucheira socialis* | Social White | Pieridae | EsCASP-3 | in house | HQ328960* |
| *Euphydryas aurinia* | Marsh fritillary | Nymphalidae | EaCASP-3 | Genbank | HM234681* |
| *Galleria mellonella* | Greater Wax Moth | Pyralidae | GmCASP-3 | in house | HQ328961* |
| *Helicoverpa armigera* | Cotton Bollworm | Noctuidae | HaCASP-3 | in house | HQ328962* |
| *Heliothis virescens* | Tobacco Budworm | Noctuidae | HvCASP-3 | in house | HQ328963* |
| *Lymantria monacha* | Black Arches | Lymantriidae | LmCASP-3 | in house | HQ328964* |
| *Mamestra brassicae* | Cabbage Moth | Noctuidae | MbCASP-3 | in house | HQ328965* |
| *Ostrinia nubilalis* | European Corn Borer | Crambidae | OnCASP-3 | Genbank | GH987442 |
| *Spodoptera exigua* | Beet Armyworm | Noctuidae | SeCASP-3 | in house | HQ328966* |
| *Spodoptera littoralis* | African Cotton | Noctuidae | SlCASP-3 | in house | HQ328967* |
| **Caspase 4** | | | | | |
| *Bombyx mori* | Silkworm | Bombycidae | BmCASP-4 | Kaikobase | HQ456874* |
| *Colias eurytheme* | Orange Sulphur | Pieridae | CeCASP-4 | in house | HQ328968* |
| *Galleria mellonella* | Greater Wax Moth | Pyralidae | GmCASP-4 | in house | HQ456875* |
| *Helicoverpa armigera* | Cotton Bollworm | Noctuidae | HaCASP-4 | in house | HQ328969* |
| *Heliothis virescens* | Tobacco Budworm | Noctuidae | HvCASP-4 | in house | HQ328970* |
| *Lymantria dispar* | Gypsy moth | Lymantriidae | LdCASP-4 | in house | HQ328971* |
| *Lymantria monacha* | Black Arches | Lymantriidae | LmCASP-4 | in house | HQ328972* |
| *Mamestra brassicae* | Cabbage Moth | Noctuidae | MbCASP-4 | in house | HQ328973* |
| *Manduca sexta* | Tobacco hornworm | Sphingidae | MsCASP-4-1a | Genbank | HM234676* (variant a) |
| *Manduca sexta* | Tobacco hornworm | Sphingidae | MsCASP-4-1b | Genbank | HM234677* (variant b) |
| *Manduca sexta* | Tobacco hornworm | Sphingidae | MsCASP-4-2 | Genbank | HM234678* |
| *Ostrinia nubilalis* | European Corn Borer | Crambidae | OnCASP-4 | Genbank | GH998299 / GH991688 |
| *Plutella xylostella* | Diamondback moth | Plutellidae | PxCASP-4 | Genbank | BP937230 |
| *Pontia daplidice* | Bath White | Pieridae | PoCASP-4 | in house | HQ328974* |
| *Spodoptera exigua* | Beet Armyworm | Noctuidae | SeCASP-4 | in house | HQ328975* |
| **Caspase 5** | | | | | |
| *Bombyx mori* | Silkworm | Bombycidae | BmCASP-5 | Kaikobase | HQ328976* (variant a)<br>HQ328977* (variant b) |
| *Danaus plexippus* | Monarch butterfly | Nymphalidae | DpCASP-5 | Genbank | EY269609 |
| *Heliconius erato* | Red Postman | Nymphalidae | HeCASP-5 | Genbank | EL603926 |
| *Helicoverpa armigera* | Cotton Bollworm | Noctuidae | HaCASP-5 | Ha Genome assembly | HQ328978* |
| *Pieris rapae* | Small Cabbage | Pieridae | PrCASP-5 | in house | HQ328979* |
| **Caspase 6** | | | | | |
| *Bombyx mori* | Silkworm | Bombycidae | BmCASP-6 | Genbank | AB292816 |
| *Galleria mellonella* | Greater Wax Moth | Pyralidae | GmCASP-6 | in house | HQ328980* |
| *Heliconius erato* | Red Postman | Nymphalidae | HeCASP-6 | Genbank | DT664351 |
| *Helicoverpa armigera* | Cotton Bollworm | Noctuidae | HaCASP-6 | PCR | HQ328981* |
| *Heliothis virescens* | Tobacco Budworm | Noctuidae | HvCASP-6 | in house | HQ328982* |
| *Manduca sexta* | Tobacco hornworm | Sphingidae | MsCASP-6 | Genbank | HM234679* |
| *Spodoptera exigua* | Beet Armyworm | Noctuidae | SeCASP-6 | in house | HQ328983* |
