# Supplementary material for: A comprehensive characterization of the caspase gene family in insects from the order Lepidoptera
Source: BMC Genomics. 2011 Jul 8;12:357. doi: 10.1186/1471-2164-12-357 (PMC3141678; doi:10.1186/1471-2164-12-357)
Supplement: Additional file 4 — Figure S3. Amino acid alignment of Lep-Caspase-3 sequences. [file 1471-2164-12-357-S4.PDF]

Figure S3. Amino acid alignment of Lep-Caspase-3 sequences. Identical residues are boxed in grey. Critical amino acids involved in substrate-binding are boxed in purple. Critical amino acids involved in the active site, including the catalytic cysteine residue, are boxed in red. Blue frames indicate putative cleavage sites.

```
                           10        20        30        40        50        60        70        80        90       100
                   ....|....|....|....|....|....|....|....|....|....|....|....|....|....|....|....|....|....|....|....|
Ac-Caspase-3       ---MAESDVDAGSYERNVGNSSVNV---------EIRTKPSFSPSD---IYYDMTGKKYLHIFNHYQYQKTRYFNNRRPSTREGTNKDVNRLTEVFGNLG
Bm-Caspase-3/a     ME---ETSNDFKIFNANPSNSSNKE------VMDNSEETQPTVSFDPESVYYNMSGDKYLIILNHYTFKKPKYSQYKQPSTRNGTFEDVKSLKDLFEGLS
Bm-Caspase-3/b     ME---ETSNDFKIFNANPSNSSNKE------VMDNSEETQPTVSFDPESVYYNMSGDKYLIIFNHYTFKKTKYFQYKQPSTRNGTFEDVKSLKDLFEGLS
Bm-Caspase-3/c     ME---ETSNDFKIFNANPSNSSNKE------VMDNSEETQPTVSLDPESMYYNMSGDKYLIIFNHYTFKKTKYFQYKQPSTRNGTFEDVKSLKDLFEGLS
Es-Caspase-3       ME---ESRADATVTDKQETNSSFPL---------VTRTRPSFNPND---LYYYGSGKKYLYIFNHYTFQTTKFFNDKQPSMRNGTSIDAIRLPQVFSELG
Gm-Caspase-3       MDDSDASPFGFLSPKAKTSDQSTEASPTENQASDRNVEVLIKRQFDPNALYYDMSGKKYLLIFNQYKFQSTVYFRNKRPSSRLGTFKDVDKLNKVFKSVQ
Ha-Caspase-3       MENMDETSSDIKGFNLNPSNTSNPI-------FVPEDE-G--LKFDKDALYYNMSGEKYLIILNHHTFKKTRYFKNQQPSTRNGTDNDVKSLKTLFGNLG
Hv-Caspase-3       MENMDETSSDIKGFNLNPSNTSNPI-------FVPEDE-G--LKFDKDALYYNMSGEKYLIILNHHTFKKTRYFKNQQPSTRNGTDNDVKSLKTLFGNLG
Lm-Caspase-3       ----------------------------------------------------------------------------------------------------
Mb-Caspase-3       MENMEETSPDVKGFNLKPSNTSNPQ-------LVP-DE-G--LKFDKDALYYDMSGEKYLIILNQHTFKKTIFFKNQQPTTRNGTDNDVKSLKKVFGNLG
On-caspase-3       MD---LTQSDARTFQSATNLLPEKSN-LPPSASRSVPLPDSSIKFDPDGLYYDMSGKKEILIFNHSEYEANEFYGKKPPDERTDHLADYERLEKVFSFFG
Se-Caspase-3       MENTGESSADVKGFNLNPSNTSNPL-------LVP-DEVG--VKFDPNALYYDMSGDKYLIILNQHTFKKTIYFQNKQPSTRTGTDNDVKSLKTIFGNLG
Sl-Caspase-3       MENTDESSADIKGFNLNPSNTSNPL-------LIP-DEVS--VKFDPNALYYDMSGDKYLIILNQHTFKKTIYFQNKQPSTRNGTDNDVKSLKAIFGNLG

                          110       120       130       140       150       160       170       180       190       200
                   ....|....|....|....|....|....|....|....|....|....|....|....|....|....|....|....|....|....|....|....|
Ac-Caspase-3       F---KVIIYNDKEHGEIIDTVTEISTRDHTETSCLFFAFLTHGDKRG--RLARWPT---------------------------------------------
Bm-Caspase-3/a     Y---NVTTYTDLIYEDILSRVAEFCKRDHSKTSCVIVTTLTHGD-SGEVFAADQPYKISDLTNMIENAHHTLVGKPKIFFIQACRGPFMDEGRTVVLDGE
Bm-Caspase-3/b     Y---NVTTYTDLIYEDILNRVAEFCKRDHSKTSCVIVTILTHGD-SGEVFAADQPYKISDLTNMIENAHHTLVGKPKIFFIQACRGPFMDEGRTVVLDGE
Bm-Caspase-3/c     Y---NVTTYTDLIYEDILNRVAEFCQRDHSKTSCVIVTILTHGD-SGEVFAADQPYKISDLTNMIENAHHTLVGKPKIFFIQACRGPFMDEGRTVVLDGE
Es-Caspase-3       F---EVETLNDKTHRQILDKFQAISSKDHTETCYLFFAFLSHGDNIGNIYAADRPYQFKDVLTFLEHGHLTLIAKPKVFFVQACRGGLTDVGRRIEMDSN
Gm-Caspase-3       F---EVSTHTDLQYTNIKETVTAFIDRDHSNTSCICITFLTHGNNQGDLYAADRPFSLQNLTKLFE--DKRLATIPKLFFIQACRGDDIDDGNFVETDAL
Ha-Caspase-3       F---EVIVFRDLEYEEIIKNLKAIATKDHSRTSCMCVTILSHGDKGGEVHAADRPYLLTDVFTIFE-KQPGLVNKPKLFFVQACRGASVDAGNTVALDG-
Hv-Caspase-3       F---EVIVFRDLEYEEIIKNLKAIATKDHSRTSCMCVTILSHGDKGGEVHAADRPYLLTDVFTIFE-KQPGLVNKPKLFFVQACRGASVDAGNTVALDG-
Lm-Caspase-3       -------------------------------------------DKGGQLYALDRPYLLNDIITIFE-KQRSLVNKPKLFFVQACRDDNLDASNTVKLDSE
Mb-Caspase-3       FKDQNIIVFRDLEYEGIIKNLTAIATQDHSKTSCICVTILTHGDKGGEVHAADRPYLLTDVIAIFE-KQRSLVNKPKLFFVQACRGGSMDAGNTVSLDSQ
On-caspase-3       F---KPFYYENKTYDVIKRTIKAKAEEDHSKTSCLAVAIMTHGLQRGILCAYDTTYYLAEMVWLLENGHSSLVHKPKLFFIQACRGWKVDPGKMTHCDGP
Se-Caspase-3       F---EVIVLKDLEYSDIIKNLTAIASKDHSKTSCICITILTHGDKGGEVHAADRPYLLTDIMAIFE-KQLSLVNKPKLFFVQACRGANMDAGNTVALDSQ
Sl-Caspase-3       F---EVIVHRDLEYSDIIKNLTAIASKDHSKTSCICITILTHGDKGGEVHAADRPYLLTDIMAIFE-KQLSLVNKPKLFFVQACRGANMDAGNTVALDSQ

                          210       220       230       240       250       260       270       280       290       300
                   ....|....|....|....|....|....|....|....|....|....|....|....|....|....|....|....|....|....|....|....|
Ac-Caspase-3       ----------------------------------------------------------------------------------------------------
Bm-Caspase-3/a     SRGVLRLPTHSDFLFLHSSVD---------------------------GYLSYRDSRGSWLIQTLCEIIRKNHKEIDLLHMITMVNKSIAYAKSTYTPK
Bm-Caspase-3/b     SRGVLRLPTHSDFLFLHSSVD---------------------------GYLSYRDSRGSWLIQTLCEIIRKNHKEIDLLHMITMVNKSIAYAKSTYV--
Bm-Caspase-3/c     SRSVLRLPTHSDFLFLHSSVDELVTRVPVLSTISRLFAVFSFKKPRKRRGYLSYRDSRGSWLIQTLCEIIRKNHKEIDLLHMITMVNKSIAYAKSTYTPK
Es-Caspase-3       HRVSYVVPTHADFFIMYSTVQD---------------------------HKSYRNKYGSFFIQDLCDIVEDYHEFYDLLHLVTLVQNRVAFLPHHARPY
Gm-Caspase-3       SFKTYHIPSHIDFLTLYPTVEG---------------------------LVAFRDREGSWMIQELCKIIETHYRDYDILQLITLANKAVAFERTSNNQK
Ha-Caspase-3       -EASFTVPTHADFLVLRSSVED---------------------------YLSYRDANGSWMIQALCHVVQKHYEDLDLLHMITLVNRHVAYERTTYTPS
Hv-Caspase-3       -EASFTVPTHADFLVLRSSVED---------------------------YLSYRDANGSWMIQALCHVVQKHYEDLDLLHMITLVNRHVAYERTTYTPS
Lm-Caspase-3       YDSVLSIPTHADFLILHSTVK-----------------------------AYRKYNGSWMIQALCDIIGKHYEDLDLLHMITLMNKKVTYER--VTPT
Mb-Caspase-3       SDSVLTIPTHADFLVLCSTVED---------------------------YLSYRDMNGSWMIQALCHIIHTITR-PDLLHMITFMT-VWCIRKSTYTP-
On-caspase-3       NSTVLHVPRHVDFFIGRSTV--------------------------------------------------------------------------------
Se-Caspase-3       SDSVLTIPTHADFLVLCSTVED---------------------------HLSYRDMYGSWMIQALCHVMEEHHEDLDLLHMITLMNRKVAYERSTYTPS
Sl-Caspase-3       SDSVLTIPTHADFLVLCSTVED---------------------------HLSYRDMYGSWMIQALCHVIKEQHEDLDLLHMITLTNRKVAYERSTYTPS

                          310       320
                   ....|....|....|....|....|
Ac-Caspase-3       -------------------------
Bm-Caspase-3/a     CQETNNKKQMTETRFTLTKLFMFEK
Bm-Caspase-3/b     -------KQMTETRFTLTKLFMFEK
Bm-Caspase-3/c     CQETNNKKQMSETRFTLTKLFMFEK
Es-Caspase-3       QNGPPQQEADARDQIHTN-------
Gm-Caspase-3       NLSIHEKKQTLETKHTLTKFLKFA-
Ha-Caspase-3       NRAMNNKKQMPETRFTLTKLLKF--
Hv-Caspase-3       NRAMNNKKQMPETRFTLTKLLK---
Lm-Caspase-3       NEDTNNKQQMIETKFTLTKYLKFQ-
Mb-Caspase-3       FYMQNNKKQMPETRFTLTKLWFDK-
On-caspase-3       -------------------------
Se-Caspase-3       NKAMNNKKQMPETRFTLTKLLKF--
Sl-Caspase-3       NRAMNNKKQMPETRFTLTKLLKF--
```
