# Supplementary material for: A comprehensive characterization of the caspase gene family in insects from the order Lepidoptera
Source: BMC Genomics. 2011 Jul 8;12:357. doi: 10.1186/1471-2164-12-357 (PMC3141678; doi:10.1186/1471-2164-12-357)
Supplement: Additional file 5 — Figure S4. Amino acid alignment of Lep-Caspase-4 sequences. [file 1471-2164-12-357-S5.PDF]

Figure S4. Amino acid alignment of Lep-Caspase-4 sequences. Identical residues are boxed in grey. Critical amino acids involved in substrate-binding are boxed in purple. Critical amino acids involved in the active site, including the catalytic cysteine residue, are boxed in red. Blue frames indicate putative cleavage sites.

```
                         10        20        30        40        50        60        70        80        90       100
                 ....|....|....|....|....|....|....|....|....|....|....|....|....|....|....|....|....|....|....|....|
Bm-caspase-4     -----MDSDENQLQTNSKTKNKRKKYLKRSKEVE--------YDLKDAVEA--------------FDEALELTENEQDNVTSDE----------------
Ce-Caspase-4     -------------------------------------------------------------------------KNKPVEMNDSEFLAPIYRPLKVYRSDS
Gm-Caspase-4     ----------------CVFNRHRSYIIIDISKYKVIEETVLFPEEIETMDANNENSEGTEDNITLLYDSIRFKSGQRMN---------------------
Ha-Caspase-4     ----------------------------------------------------------------------------------------------------
Hv-Caspase-4     ---------------------------------------------------------------------------------SEEVTYRDIVTARDSSNTA
Lm-Caspase-4     ---MERDIYETYAEYQAVCRKSKKKKRQRSKQFS--SPEEFRTESNSQLEE--------------LANESPVNDDNVSEEESDDVTTKNVFNKLEKQANP
Mb-Caspase-4     MENDNTGYYPNYSGFQFLLKKNRGKKKKRGKKTKTHNITGYTKSDAGTLEL--------------LDD-ATLTDDN-SG--DTEEGSRDLVTLRANEDDA
Ms-Caspase-4-1   --MDSELQDTSLQKTETELKK-KRKRRNRGKQTRMSDDVDNGSSDSNVVEADALDNANTEEIIDLMTNKWEIDDSPRVNNDINENTEYYETSIDTIHSQS
Ms-Caspase-4-2   --MEGENKGTNIQNQGTELQK-KRKRKKRGKQKRISEDVDNGSCVSNIVEVDAPDNANTVENIDLITNKSEIDENPRFDKNTNENTEYHEATTDTLHSQS
On-Caspase-4     ---------------------------------RGSGKPVDGDNTPNDGCSNAGDSENEGNERVNIIYGVPINNSDEESLLDTD----------------
Pd-Caspase-4     -----------------------------------------------------------------------KSKPFFKIKSKRDFPKLKHMDNNSDSDDD
Px-Caspase-4     ----------------------------------------------------------------------------------------------------
Se-Caspase-4     --------------------RQKGPTIMGDKQNE--------KSESETLES--------------VEDPSNLTDENGSGLDETEDDSNKSVIYRANDGES

                        110       120       130       140       150       160       170       180       190       200
                 ....|....|....|....|....|....|....|....|....|....|....|....|....|....|....|....|....|....|....|....|
Bm-caspase-4     -----AVESVFVDAIMKTPPKLL-------HPQKVIYPE------------------------PVEIPPVETKNIEEKRVS--DSK--ITDQVEEIESES
Ce-Caspase-4     -----EEDNITIDADLKKPPEY--------------------------------------------EEKEIEKEEEELEVECLDNN--------------
Gm-Caspase-4     -----RFNNVPRDV-------------------------------------------------DTSTVETEKQEVQQKNVR-------------------
Ha-Caspase-4     -----AENRVSLDAMMNAPP----------EYAKSEEV-------------------------MTAELKTLESSLEKLNME--DES--VFTESSEPKMEY
Hv-Caspase-4     VNF--SENRVSLDGMMSAPP----------RYENSEAV-------------------------ITAELKTLENSLDKLHME--EES--VF-ENSEPKMEY
Lm-Caspase-4     ENVYPSGSHVYRDVMVYAPPVIRATPEEEKSLANQEFY-------------------------YSSENLGSKGTIKIIHGEPIEEQ--ASEETS------
Mb-Caspase-4     VFN--DENRVSLDAMMSVAP------EYE-FETKVHVV-------------------------ENAELP--ESSVDKLNLE--DEAFDTI-ESIDSKRQY
Ms-Caspase-4-1   PSRVETGQRFFSDANNDD--EYYRRKDANLTADNNVNIFTGVSQKTNNVFTTYDGIASSCVTSNENITKMEKSSITRLTSEDVEKL--YPSTSSATETVP
Ms-Caspase-4-2   SSQVKTCEHLFSYSNNNDPPDYEHLKDENLSADIKNNIV------VQNLVT--DGIPSTCENPSTKVLHDGNKLSTQLSSESDTKL--NRSISSTTKNLI
On-Caspase-4     -GVLASSSTLVLDAQMKKPP----------------------------------------------VLPLEDKKEDIDYIEYVREE--SLKEHEETH--E
Pd-Caspase-4     AQN--IDNAITTDGEVFNPPCY-----------------------------------------EGEATETKETPIEEKNIIYPNTL--------------
Px-Caspase-4     ----------------------------------------------------------------------------------------------------
Se-Caspase-4     ADL--NENRISIDAMMSAPP------EYEKSQEKVKFV-------------------------DNEDLETLESNVDKLQIE--EES--VF-ENEDTK--Y

                        210       220       230       240       250       260       270       280       290       300
                 ....|....|....|....|....|....|....|....|....|....|....|....|....|....|....|....|....|....|....|....|
Bm-caspase-4     KFYNNRALTKYATTYELEKFEKNALLIFNQENIYGY-SPRKGTEKDVESLTVTFQKFGFEVVEHKDLTKDEVLNEIKEFSSRDLRDYGCIGIAVLTHGSS
Ce-Caspase-4     -IFNQRALSKYATTYELEKFEKNLLIIFNQENIYGY-KPRLGTEQDVRELENTFSRFGFVVEEYKDYTKEEIFQVLDTFKRRDFTDYGCVAIAVLTHGTR
Gm-Caspase-4     ---KRKVLSRTALTYELEKFELKILLIFNHIKVINH-EERRGTEEDVKSLKCTFGNFGFTVTEYVDLTEKQIKNTLKDFTSKNLSNYGCIAVAVLTHGSY
Ha-Caspase-4     VI-NKRALPKEATTYELEKFEKNALIIFNQENIEGH-QRRLGTEQDVEALTSTFGSFGFEVTPHKDLTHDELFKELKAFSEKDFTDYGCVAVAVLTHGSN
Hv-Caspase-4     VI-NKRALPKEATTYELEKFEKNALIILNQENIDGH-QRRLGTEKDVEALTNTFENFGFEVTPHKDLTHDDLFKELKAFSEKDFTDYGCVAVAVLTHGSN
Lm-Caspase-4     LL-NTKALSKFAMTYELEKFQKNALVIFNQENVDGH-QRRLGTERDVESLTKTFENFGFEVVEHKDLTKDEVFKELKTFTDRDFTDYGCVAVAVLTHGSN
Mb-Caspase-4     SL-NTRALPKEATTYELETFDKHALIIFNQKEIDGH-QPRLGTEKDVDAMTKTFKNFGFEVTTHDNLTKDGIFDELKTFSERDFTDYGCVAVTILTHGSN
Ms-Caspase-4-1   QLYNARALPKEAKTYELENFDRNVLIIFNQKDFGTH--QRLGTDQDVKALKKTFEAFNFDVLPYTDRTVAQIKEILDAISCQDFSDYGCVAIAVLTHGDI
Ms-Caspase-4-2   QLYNPRALPKESETYELEKFDKNKLIIFNQIKFGQY--ERLGTEADLKSLKTTFEAFNFEVQDYPDPTVDKIEEILNEISVTDYSDYGCLAIAVLTHGGI
On-Caspase-4     IVYNKRALSKEAPTYELEKFGKHALLIFNQCNIEGYVRPRLGTERDVEELEKTFKSFGFEVETVPDLTKEEIRTKLKEFSSRDFTDYGCVAVAVLTHGSR
Pd-Caspase-4     -WRNTRPLSKYATTYELEKFEKNCLIIFNQENVIGY-EPRLGTDKDVKILSDTFSRFGFDIEKHKDLTKRELFRVLDSFNYRNFSEYGCVAVVVLTHGTI
Px-Caspase-4     ----------------------------------------GT----------------------------RVENKLKEFSEKDFTDYGCVAVVVLTHGSD
Se-Caspase-4     TLINTRALPKEATTYELEKFTKHAMIIFNQKEIDGH-LPRLGTEKDVEALTRTFSNYGFEVTPHNNLTKDELFKELKTFSERDFTDYGCVAVAILTHGSN

                        310       320       330       340       350       360       370       380       390       400
                 ....|....|....|....|....|....|....|....|....|....|....|....|....|....|....|....|....|....|....|....|
Bm-caspase-4     DGMLRAKDMQ-YSEKEILRHFKVHDKPFLVTKPKFLIIQACRGKEEMQGAAVF-RS-GK----LRKDLD-E---DLEPYVLPIEADMLILHSSYSGKPSH
Ce-Caspase-4     DGILRARDQQ-FTEFDIIDALKTHNKPTLITKPKLLIIQACRGSKDIQGVIVG-RS-VK----IRKDIDD----DFNPYTLPVEADMFVLHSSYMGNPSH
Gm-Caspase-4     DGELMAADTS-YNEKMIVEYLKAGDNNTLITKPRIVIIQACRGKGDIKAVTVSGTEEND----IFTD----AINESESYTLPMEADILVLHSCYEGNPAH
Ha-Caspase-4     NGLLRAKDQQ-YSEIKIINHFKDYSKSTLVTKPKLIIIQACRGTKIKEGVPVF-RS-GK----IRKDLDEE---DLEPYILPVESDLLILHSSYVGSPSH
Hv-Caspase-4     NGLLRAKDQQ-YSEIKIINHFKDYSKSTLVTKPKLIIIQACRGTKIQEGVPVF-RS-GK----IRKDLDEE---DLEPYILPVESDLLILHSSYVGSPSH
Lm-Caspase-4     DGLLRARDQQ-YSEIEIINYFKVLNRPTLVTKPKIIIIQACRGTRIVPGTPVF-LS-GK----VRKDVDDE----LEPYILPVEADMLILHSSYGGSPSH
Mb-Caspase-4     NGMLRAKDQL-YSEIEVINHFKDYSKPTLVTKPKIFIIQACRGTKIMTGMPVF-HS-GK----IRKDLDEE---DLEPYILPVESDLLILHSSYPGRASH
Ms-Caspase-4-1   DGSLSAKDDD-YPEMDIINTLKTHKNHSLITKPRLLFVQACRGPNALAGVPVL-RK-GKVKSNLFYD--------SKPYTLPVEADMLVVHSSYTGNRTH
Ms-Caspase-4-2   DGKLLAADGD-YYEIKIINFLKTHKNHSLITKPRLLFVQACRGPNSLIGVPVL-QP-GKVQSNLIYD--------TEPYTLPVEADMLVVHSSYVGNRAV
On-Caspase-4     NGLLRAKDTD-YSELTIINQLQVHDKPTLVTKPKLVIIQACRGYGSMQGTTVR-RAQGQ----IMKDLDEE-----EPYTLPVESDMLVLHSSYIGKPSV
Pd-Caspase-4     DGRLRAKDQH-YTEFDVIDAFKTHDKPSLITKPKLLIIQACRGKKSVQGVAVG-LSSNA----IRKDEI-Y---DFEPYTLPVESDMFVLHSSYMG----
Px-Caspase-4     MGRLKTSDNKVIYEAEIIQYFNNRN-PTLVTKPILFIIQACRGTKMSEAILAG-RV-SE----MTKDADEE---EIVPYTVPAESDMLVLHSSYVGKLSI
Se-Caspase-4     NGLLRAKDQQ-YSEIEVINHFKDSSKPTLVTKPKIIIIQACRGTKITQGLPVF-HA-GK----IRKDVDEE---DLEPYILPVESDLLILHSSYVGRASH

                        410       420       430       440       450       460       470       480       490       500
                 ....|....|....|....|....|....|....|....|....|....|....|....|....|....|....|....|....|....|....|....|
Bm-caspase-4     RHEVDGSWFIQSLCSKINELSETHDLESIITEVKREVAIDRYHKEFNRRTSEYDINKQMPVITSTLIRKLYLRKY-MDPPVSQCLIKPT--PAISESSKN
Ce-Caspase-4     RDEINGSWLIQTLCHKIKLLSSTHDLEHIIIDVKREVAIDKYHEEYNRRTLEIDTNKQIPVVTSTLIRKLYFRRFGEENNAVSHTDSNL------VQSKN
Gm-Caspase-4     RTD-KGSWFIKTLCEEIDRLAATEDLESIITVVKRRVAIDYIHEKYDSIKTEYSYNKQMPVSTSTFTRKLYLRRYK------------------------
Ha-Caspase-4     RNELHGSWFIQALCNKINALSTTHDLESIITEVKREVAINKQHEEYNRRTFEMDINKQMPVMTSTLIRKLYLKRF-GDIPK---TPTFVDRARRSSDSKN
Hv-Caspase-4     RNELHGSWFHQALCNKINALSTTHDLESIITEVKREVAINKQHEEYNRRTFEMDITKQMPVITSTLIRKLYLKRF-GD----------------------
Lm-Caspase-4     RNELHGTWFIQALCNKINTLAPTEDLESIITEVKRDVAINKQHEEYNKKTLQLDINKQMPVTTSTLIRKLFLRKF-GDGSNPQYTLLDTKRESISCESKN
Mb-Caspase-4     RNELYGSWFIQTLCKKIDTLS-------------------------------------------------------------------------------
Ms-Caspase-4-1   RHEVDGSWFIQTLCKKINEMGQTDDLETILTHIKHEVAIDKEHEVYNRVTGEYDINKQMPVVTTTLIRKLYLRRDAAEGTVTSNTSEMPGAIAVDSH---
Ms-Caspase-4-2   RHQREGSWFIQTLCKKINEMASTHDLETILTHIKREVASDKEKIIVNRVTKEHEINKQMPVVTTTLIRKLYLKRSAAEENAL-NTNGMPTDVAVDSY---
On-Caspase-4     RDELEGSWFIQSLCKKINELASTHDLESIMTEVRREVAIDRVHEELNRRTDEMDVIKQMPVATSTLIRKLYLKNPVGDSPGPSSTLTTD-----SDQPPR
Pd-Caspase-4     ----------------------------------------------------------------------------------------------------
Px-Caspase-4     RHELG-----------------------------------------------------------------------------------------------
Se-Caspase-4     RNELYGSWF-------------------------------------------------------------------------------------------

                        510       520       530       540       550       560       570       580       590       600
                 ....|....|....|....|....|....|....|....|....|....|....|....|....|....|....|....|....|....|....|....|....
Bm-caspase-4     EVLDESTPDTN--LLVQCGQCTCYLDHFLYMKSCLEYYLEVRPD--DETALNFLKVANTFQHLDEFNSSKEQMVTAISKYLMKNAEGMHFYKYK----------
Ce-Caspase-4     EVLDASSP-----VSLEFGPCLCFLDYYAYIKNCLRLFLDKNPE--DATAQSFFDISESFDDNFEFNSAKEKMTKAICNHLSIYAKDFEFYKYLYIKTTVDRLK
Gm-Caspase-4     EVSEQTEVS--------------------------------------------------------------------------------------------
Ha-Caspase-4     QVLDASMASSPGTPLIQFGPCNCFLDHFTYMRNCLRYFVEENPH--DVAGQSILDVANTFEDGVE---------------------------------------
Hv-Caspase-4     --------------------------------------------------------------------------------------------------------
Lm-Caspase-4     EVHDNAIPTTP--LLIQFGPCSCFLDHYNYMRTCLQYFIDENPN--DEMAQNFLEIANTFEEGVEFNTAKAKMSKAIGKYLMSKTRSCQYFKYLYFYKH-----
Mb-Caspase-4     --------------------------------------------------------------------------------------------------------
Ms-Caspase-4-1   --------NAGANP----EPCLCTLDYYNYIRDCLREYVRDHDQ--DTTARSLLDLTKTFKDEETFNATKEQCALTIADHLEHSVKHYERYKFVYIKRI-----
Ms-Caspase-4-2   --------NTGANPKPELENCRCNLNYFDYIKECLRHFLQDNNDYKDTTGRSLLNLTDTLKNEEAFNATKEKCAMTIADYLETRAKHCQHYKYIYINKK-----
On-Caspase-4     PFLDNSVPNTP--LLVQFGPCSCFLEHFNYIIDCLRHFLNDNPD--DVLAQSFLEMADTFEDSANFNSSKEKLAKAVCAYLKSQVN------------------
Pd-Caspase-4     --------------------------------------------------------------------------------------------------------
Px-Caspase-4     --------------------------------------------------------------------------------------------------------
Se-Caspase-4     --------------------------------------------------------------------------------------------------------
```
