# Supplementary material for: A comprehensive characterization of the caspase gene family in insects from the order Lepidoptera
Source: BMC Genomics. 2011 Jul 8;12:357. doi: 10.1186/1471-2164-12-357 (PMC3141678; doi:10.1186/1471-2164-12-357)
Supplement: Additional file 6 — Figure S5. Amino acid alignment of Lep-Caspase-5 sequences. [file 1471-2164-12-357-S6.PDF]

Figure S5. Amino acid alignment of Lep-Caspase-5 sequences. Identical residues are boxed in grey. Critical amino acids involved in substrate-binding are boxed in purple. Critical amino acids involved in the active site, including the catalytic cysteine residue, are boxed in red. Blue frames indicate putative cleavage sites.

```
                         10        20        30        40        50        60        70        80        90       100
                 ....|....|....|....|....|....|....|....|....|....|....|....|....|....|....|....|....|....|....|....|
Bm-Caspase-5/a   MQEEHKKAIQRNFSSLVEGTDLDSMVMALYEKGVFSQQMIEPYRDTSITPRERKRILYREITRRGPQAFTMLLEALREMGHWDLVRDLDPNSPLQL-RA-
Bm-Caspase-5/b   MQEEHKKAIQRNFSSLVEGTDLDSMVMALYEKGVFSQQMIEPYRDTSITPRERKRILYREITRRGPQAFTMLLEALREMGHWDLVRDLDPNSPLQL-RA-
Dp-Caspase-5     MEERHRRAIQQNFTSLVEQTDLDMMVSALYERGVFSEQMIEQYKDVSKDVRYRKRLLYMDVTRRGPQAFGHLLDALFEAGHWNLVRDLDPHSA-LHSRPQ
Ha-Caspase-5     MEQKHKEAIRSNFSSLVERTDLDSVVTSLYEKGVFSEPMIEPYRIISLPERDRKRQLYVDILRRGPLAFGHLVDTLGELGYWDLVRDLDPDSPFSFPGQR
He-Caspase-5     MQEEHRKAIQNNFKSLVEQTDLDTMVTNLYEKGVFSSQMIEKYKDTNKDVRNRKRLLYSDIMRRGPEAFQHLLDALMENGYWDLIRDLDPKSLPPH----
Pr-Caspase-5     ----------------------------------------------------------------------------------------------------

                        110       120       130       140       150       160       170       180       190       200
                 ....|....|....|....|....|....|....|....|....|....|....|....|....|....|....|....|....|....|....|....|
Bm-caspase-5/a   --RRPVLPPNL----SGPQDN-FVSLTIEKH--KP-K-----NNNNDIIKPRPGSDAPVPPPP-VNSSEP-------V-AIPCF-HVKESTHFFEDDV
Bm-caspase-5/b   --RRPVLPPNL----SGPQDN-FVSLTIEKH--KP-K-----NNNNDIIKPRPGSDAPVPPPP-VNSSEP-------V-AIPCF-HVKESTHFFEDDV
Dp-Caspase-5     TTRR--PLPDTSSHSS---ENNFVSISAQK---KKTKSVPEPAKSPRRHTPATSS----PG-P----DEAED-----TAEIPQD-VVK-STKFYEDG-
Ha-Caspase-5     TF-N--PIPKLNPPTTG-NDN-FHSLSRPKGDV---------NKNPRQSS-------P-PPAPAPHQSSDNDNAACDVTIEPPFTVVK-STKFMEDND
He-Caspase-5     SRRRT-PLPAT-SKSSG--DNNFVSIR--KEREKKAKSAPDVNKNVGN--I------P-PAPP-----------------------------------
Pr-Caspase-5     --------------------------------------------------------------------------------------------------

                        210       220       230       240       250       260       270       280       290       300
                 ....|....|....|....|....|....|....|....|....|....|....|....|....|....|....|....|....|....|....|....|
Bm-caspase-5/a   SRDIQLYRTRSRKRGVLVVFSYISFLLNIEPNRDGVNVDCDKLKYLFSEIGFEVLTYQNLTLEQTRATLEQMSSVLAGYECVFLVVSSHGHERRYSSD
Bm-caspase-5/b   SRDIQLYRTRSRKRGVLVVFSYISFLLNIEPNRDGVNVDCDKLKYLFSEIGFEVLTYQNLTLEQTRATLEQMSSVLAGYECVFLVVSSHGHERRYSSD
Dp-Caspase-5     -HDIKLYRTRGRNRGVLVVFTYIEFLHNTASYRRGADVDCQRLKYLFNELGFKVLSYINLTESETWNTLHSLKEVVAGVDC-----------------
Ha-Caspase-5     TKDVRLYRTRGRNRGVLLDFTYMKFDNDIEEFRNGVDVDCNNLKYLFNELGFRVLRYLNLTMAETTETLRSLDAVLVDIESVFVVVSSHGYERAHSSD
He-Caspase-5     --------------------------------------------------------------------------------------------------
Pr-Caspase-5     ---------------------YIEFVNNIETYRKGVDVDCQKLKYLFSEMGFRVISYKNLTRDETVTTLKSLKKVIVGVECVFIVVSSHGYERKYSSD

                        310       320       330       340       350       360       370       380       390       400
                 ....|....|....|....|....|....|....|....|....|....|....|....|....|....|....|....|....|....|....|....|
Bm-caspase-5/a   TDIRCSDGKFISLYAIVDYFNNRKLPNFTDLPKVFIFQACRGENEDFVQGGGA------LAPDPL------------------------PLHHAEK--PL
Bm-caspase-5/b   TDIRCSDGKFISLYAIVDYFNNRK------------------------------------------------------------------LHHAEK--PL
Dp-Caspase-5     ----------------------------------------------------------------------------------------------------
Ha-Caspase-5     TDVRCKDGKLMRLMDIIDYFNNRNLPALIGVPKVFIFQMCRGSSRDHAWTDAAPAPPHMRMPDGVLYSTDGVLYDGQPVQPAAADRQ---LSRGRRELPL
He-Caspase-5     ----------------------------------------------------------------------------------------------------
Pr-Caspase-5     TDIRCKDGQLITIYEIIDYFNNKNFPALHGIPKVFIFQLCRGSNVEYIDDPIRF-----VSPDDPNSPAERDGNPMTDTGRFSQDTPRTPLSKRNR---L

                        410       420       430       440       450       460       470       480
                 ....|....|....|....|....|....|....|....|....|....|....|....|....|....|....|....|...
Bm-caspase-5/a   YSNILIANSTLPGFISHRDVKAGSWYIQVLCEVFAERAHDCHVDELFTLVDKRLRLRFGRQTSSVDRWGFNNKLYLHPGLYE-
Bm-caspase-5/b   YSNILIANSTLPGFISHRDVKAGSWYIQVLCEVFAERAHDCHVDELFTLVDKRLRLRFGRQTSSVDRWGFNNKLYLHPGLYE-
Dp-Caspase-5     -----------------------------------------------------------------------------------
Ha-Caspase-5     YSDILTAHSTLPGLVAHRDGKFGSWYIQVLCKVFAERAHDCHVEKLFTLVDKRLQAEFKVQTSSVDRWGFNKRLYLHPGLYES
He-Caspase-5     -----------------------------------------------------------------------------------
Pr-Caspase-5     FSDILIAHSTLPGNVSYRDPEEGSWYIQTLCEVFAARAHDCHVDKLFTLVDKRLHERFHIQTSSVDKWGFNKRLYLHPGLYQD
```
