# Supplementary material for: A comprehensive characterization of the caspase gene family in insects from the order Lepidoptera
Source: BMC Genomics. 2011 Jul 8;12:357. doi: 10.1186/1471-2164-12-357 (PMC3141678; doi:10.1186/1471-2164-12-357)
Supplement: Additional file 7 — Figure S6. Amino acid alignment of Lep-Caspase-6 sequences. [file 1471-2164-12-357-S7.PDF]

Figure S6. Amino acid alignment of Lep-Caspase-6 sequences. Identical residues are boxed in grey. Critical amino acids involved in substrate-binding are boxed in purple. Critical amino acids involved in the active site, including the catalytic cysteine residue, are boxed in red. Blue frames indicate putative cleavage sites.

```
                         10        20        30        40        50        60        70        80        90       100
                 ....|....|....|....|....|....|....|....|....|....|....|....|....|....|....|....|....|....|....|....|
Bm-Caspase-6     MFRPDALD---ERAALDRQIIHSNLINVDVISQIERELQDEPYDMVSLVFLLYEVPDTALQKLVTHQKIVTEMLG-TNLNLLHDWYQHSKSKPTWKHEFL
Gm-Caspase-6     MLKSDALTHSRWYTTGDT----YPSINIQSIIEIEKELE--PYDLISLVFLLYDVPETALQRLIIFQRVYKDIGG-SNSNLLQEWARHAQNRDMWKHQFL
Ha-Caspase-6     -MQSDSR---IQGFDVENIIGNSDVMNIDMMSEIEKELQDNPYDMISLVFLLYDTPDTALQRLIVFQRVSNDAASSINLNMLHEWLRHAKHDPNWKHQFV
He-Caspase-6     ----------------------------------------------------------------------------------------------------
Hv-Caspase-6     -FSEDAVRFPYTGIDVENIIGNSDVMNIDMMSEIEKELQDNPYDMISLVFLLYEVPDTALQRLIVFQRVSNDAACSINLNMLHEWLRHAKHDPNWKHQFV
Ms-Caspase-6     MLSSDARSSLNSKSDNE-----TFILNLDSISKIEKQLQDNPYDMISLVFLLYDVPDTALQRLMVYQRVTSDVSG-TNINLLQEWYCHASSRPDWQHELL
Se-Caspase-6     MLSLDSVNTTPQRMEVENVIGNSDITNIDMMSQIEKELQDNPYDLISLVFLLYDVPDTALQRLIIFQRVSNDASNSINLNMLHEWFRHAKHNPNWKHEFV

                        110       120       130       140       150       160       170       180       190       200
                 ....|....|....|....|....|....|....|....|....|....|....|....|....|....|....|....|....|....|....|....|
Bm-Caspase-6     EALLICQLFNIVRRIGFDVQTLRKHYQTDYPGLSMYVDPLRKILYKICEEIDTPNLIKLQKSLLTYDIDVSGHNICEIILLELMSRRFIGI-KYCRHDEK
Gm-Caspase-6     EALITCQIYNVVRKLGFNVAAVKKQYQENG---EHFINPMKKLLYKLCENIDSEKLHKLKKTLSTYSIDTTMFESCELIFLDLMCRKFIEVQQYQY-AQK
Ha-Caspase-6     EALLICQLHSVVRKLGFHVQTARKYYQTDNIAVKMYVNPMKKALYKLCESINSDNLLKLKKTLLTYDIDATEYDSCELVFLKLMCDKFITINQYQY-NSK
He-Caspase-6     ----------------------------------------------------------------------------------------------------
Hv-Caspase-6     EALLICQLYSIVRKLGFHVQTARKYYQTDNIAVKMYVNPMKKALYKLCESINSDNLLKLKKALLTYDLDTTEYESCELVFLKLMCEKFIIINQYQY-NSK
Ms-Caspase-6     EALMICQLNSIVKSLGFHIPTMRVFYQSNDPFSSKYINPVKKVLYHACENINSTNLLKLKKSLLSYDINVMEYTTCELIFLELLSNKFITINNIGQKI-K
Se-Caspase-6     EALLTCQLYSIVRKLGLNVNAARKYYQTDSITVKMYLNPMKKALYKLCESITSDNLLKLKKALLTFGIDTTEYESCEQVFLKLMCEKFIKINQYQY--SK

                        210       220       230       240       250       260       270       280       290       300
                 ....|....|....|....|....|....|....|....|....|....|....|....|....|....|....|....|....|....|....|....|
Bm-Caspase-6     -YL-TEIKIDKFLRIIENFDGLRKLSLDLKFLQNKFANETNPNNSLKC------------------KMDTTSQQNEQPVLVEHTNEGGIF-DINNLLDI
Gm-Caspase-6     -ITGNEYNIENLAKIIENFSGLEEFAKQLRDLEAMTKTEGGDLHSHPEVPPSAVNKDELTVKED---KVEDKFTHK------------MFDDTFD-L-I
Ha-Caspase-6     -VLGFKVNVDKLIKIVENLPGLRKLALEINMLQQSMTEE--PRTTIVT---STP-SQHMKVDESKQAKVDE-NY-----------YNMDFDDVFEMLG-
He-Caspase-6     ----------------------------------------------------------------------------------------------------
Hv-Caspase-6     -VLGFKVNVDKLIKIVENLPGLKKLALEINMLQHSMSDELTPRTMVVT---STP-SEHMKVDESKQEKVDK-NY-----------YNMDFDDVFEMLG-
Ms-Caspase-6     TNANCICNVENLVKILDNLNGLKKVAMNLRYFASKFNDEEVDSFASVGNGSSSPSLPVPPLDGTKL-KADDKN------------YGA--ED-FSELF-
Se-Caspase-6     -ILGSRVEVDKLVKILENLSGLKKLVLEINILQQSLNDEHKPS-VSVAT--STPIVKHM--------KVDETNQENADDNF-----YNIDFEDMFEMLG-

                        310       320       330       340       350       360       370       380       390       400
                 ....|....|....|....|....|....|....|....|....|....|....|....|....|....|....|....|....|....|....|....|
Bm-Caspase-6     HEDLEKLQINDGFHFEADKNRHMEEIYEIKNKNRLGLCLIINQENFYPSRQSIELNNQMDPLQTRTGSTKDRIALENTMSLFNFVVNSRSDLGHKEMINF
Gm-Caspase-6     NEFMGKL-TM-DG-LATDK--LNNDTYCIKNPSKVGVCYILNQEDFHPSKSSIESKGNFKPLDKRKGSTKDKLALEETMSKLNFEVITRDNLDRQTMLED
Ha-Caspase-6     -EL--KLDEMTQESLKSDRKQLDIDSYEIKSNKRVGICVIINQENFYPSKQSIEIKNGQTQLDKRIGSDKDKMVLEKTMKSLNFAVISATDIGHKAMLEF
He-Caspase-6     -------------------------------ARGKNLIGLCCVINEENFYPTKENIQLGNKVN-LSDRVGSSLDVIALEKTMKALNFVVISKHNLSRNEVFQF
Hv-Caspase-6     -EL--KLDEMTQESLKSDRKQLDNDSYEIKSNKRVGICVIINQENFYPSKQSIEITHGQTQLDKRIGSDKDKMVLEKTMKSLNFAVISATDIDHKAMLEF
Ms-Caspase-6     -DIINNMPEPLENNFKSDTMSTKQNRYEIKNPEQLGVCIVINQENFYPQKNQIEDHQI-VPLEERKGSSVDKRTLERTMTSLKFQVHSCSDLDHDEMIEF--
Se-Caspase-6    --EL--QLDEIAQNSLKSDRKLLDNDSYEIKSNKRVGVCVIINQETFYPSKTSIELNGQNIIPDTRIGSDKDKMILEKTMSELNFTVFSKTDLDHKEMMQF

                        410       420       430       440       450       460       470       480       490       500
                 ....|....|....|....|....|....|....|....|....|....|....|....|....|....|....|....|....|....|....|....|
Bm-Caspase-6     IKDNIKNNIHEDDSMFMLCILSHGVRGHIFAADSVKIKVEDIQKLLDSDEAKKLHCIPKVLIIQACQVI-ENQNPY--LTTDSPL-GNIFIKKSDFLVYWA
Gm-Caspase-6     IKNVIKYRVHKYHSMFMLCILSHGIKGHVYAADSVKVKVEEIESLLDCDEVNHLRGMPKVIVLQACQVSNEDQYQDVDLAADGPK---YYIKKSDVLIYWA
Ha-Caspase-6     IMDVVKNKVSEEDSIFMLCILSHGVRGQVYAADSVKVKMDDIQNMLDSDEAVNIHNMPKLLIVQACQVD-EASHPV--LVADSPK-DYCNLKKTNFLIYYA
He-Caspase-6     LKDVIRDKILPEDSIFMLCILSHGVIGHVYAADSIKIKVQDIQNLLDSDESHNLYDIPKVLIIQACQSEEESGANLN-IVSDGPSSRDKHLKKTNFLVFSA
Hv-Caspase-6     IMDVVKNKVSAEDSIFMLCILSHGVRGQVYAADSVKVKMDDIQNMLDSDEAVNIHNMPKLLIVQACQVD-EATHPV--LVADSPK-DYCNLKKTNFLIYYA
Ms-Caspase-6     IKKKINKHVTSNDSIFMLCILSHGVRDHVYAADSVKIKVESIQNLLDSDEMSHLRGIPKVFIIQACRV--EDTPHPT-FAADNVQTNYYL-GKLDFLIYWA
Se-Caspase-6     IMAVVKHKVHKNDSIFMLCILSHGVKGQVYAADSVKVKMDDIQNLLDSDDAANIHNMPKLLIVQACQVD-ENTPQI--LVADSPR-DYN-LRKSNFLVYYA

                        510       520       530       540       550       560       570       580
                 ....|....|....|....|....|....|....|....|....|....|....|....|....|....|....|....|....|
Bm-Caspase-6     TAPEYEAIRHITKGTVFIQNLCKAIRLYGNKKHLSDIFTIVNNTVTNYCMKLNHPQVPIYENSLRRALYLFKP---------
Gm-Caspase-6     TAPDYEAYRIEAYGSLFIQILCSVIQKYADKDHLCDLFLKVNYIVHKACTKLCCDQLPKIESTLKKKLYLKRLA----------
Ha-Caspase-6     TSPDLEAYRNEKKGSIFIQILCRTIRKFANVEHVFDIFTKVTDNVTLLCTKVGCAQVPIFQSTLRKKLYLQIPQ----------
He-Caspase-6     TVPGYEAFRDIDVGSIFIQCLCDIIRKKANHEDLFNIFTEVTDCVLSICTELKRDQVPSFESTLRKKIFLRRNNILLSYSHSWL
Hv-Caspase-6     TAPDLEAYRNEKKGSIFIQILCRTIRKFANVEHVFDIFTKVTDNVTLLCTKVGCAQVPIFQSTLRKKLYLQIPQ----------
Ms-Caspase-6     TAP-YEAFRHEQTGSLFIQALCKLLRQRAKHDHLHEIFTQVNNNVANLCTKLQRAQVPLFKSTLRKNLYLQVPE-----------
Se-Caspase-6     TAPELEAYRNEKRGSIFIQVLCRTIRKFANSEHVYDIFTKVNNNVNFIC----------------------------------
```
