# Supplementary material for: A comprehensive characterization of the caspase gene family in insects from the order Lepidoptera
Source: BMC Genomics. 2011 Jul 8;12:357. doi: 10.1186/1471-2164-12-357 (PMC3141678; doi:10.1186/1471-2164-12-357)
Supplement: Additional file 8 — Figure S7. Amino acid alignment of noctuid-derived caspase-1 and caspase-2 sequences. [file 1471-2164-12-357-S8.PDF]

Figure S7. Amino acid alignment of noctuid-derived caspase-1 and caspase-2 sequences. Identical residues are shaded in dark grey and conserved residues in lighter grey. Blue frames indicate putative cleavage sites. Note the apparent lack of cleavage site between the prodomain and the large subunit in caspase-2 sequences.

```
Sf-C1    1 -------------------------------ALGSNSSSQPNRVARMPVDRNAPYYNMNHKHRGMAIIFNHEHFDIHS-LKSRTGTNVDSDNLSKVLKTLGFKVTVFPNLK  79
Se-C1    1 MLDGK-QDNGNVDSVDTKQR--TNGGGDEGDALGSHNSSQPTRYARMPVDRNAPYYNMNHKYRGMAIIFNHEHFDIHS-LKSRTGTNVDSDNLSKVLKGLGFRVTVINNLK 107
Mb-C1    1 MLDGEPQDNG---SVDTEQRPNANGGGDEGDALGSHESSNARRYARMPVDRNAPYYNMNHKYRGMAIIFNHEHFDIHS-LKSRTGTNVDSDNLAKVLKGLGFRVTVLSNLK 107
Ha-C1    1 MLDGDVQDNG---SVETEQR--PNGGGDEGDAQGSHDSSHARRFARMPVDRNAPYYNMNHKYRGMAIIFNHEHFDIHS-LKSRTGTNVDSDNLAKVLKGLGFRVTALNNLK 105
Hv-C1    1 MLDGDVQDNG---SVEAEQR--ANGGGDEGDAQGSHNSSHSRRIARMPVDRNAPYYNMNHKYRGIALIFNHEHFDIHS-LKSRTGTNVDSDNLFKVLKGLGFRVTVLTNSK 105
Hs-C1    1 MLDGDVQDNG---SVETEQR--TNGGGDEGDAQGSHDSSHSRRLARMPVDRNAPYYNMNHKYRGMAVIFNHEHFDIHS-LKSRTGTNVDSDNLSKVLKGLGFRVTVLSNLK 105
Se-C2    1 --------------------MAH----------SSTNGSQVSCAESFNSYKN-EFYDMNHKYRGKAIIFNHDRFDID--IPPRAGSLKDCDNLEECLTALGFSVDIFHNFK  78
Mb-C2    1 --------------------MAERNEET-----------APH-PGPCNAYRN-EFYNMNYKYRGKALIFNHHHYATALELRPRGGTNKDCESLVECLKQLAFSVDVFCDLK  78
Ha-C2    1 --------------------MSEGNGVTEQ-KLENTNGTRPKCLPESNSYRN-EFYNMNHKYRGKAIICNHEHFEID--LKPRPGTGKDCENLEKCLNYLGFSVDALSNPK  87
Hv-C2    1 --------------------MSEGNGVTEQ-KLKSSNGTRPKCSTTFNSYRN-EYYNMNHKYRGKAIIFNHEYFEID--LNPRPGTGKDCENLEKCLKDLGFSVDVLCDPK  87
Hs-C2    1 --------------------MSEGNGVTEQKKLKSSNGTSPKCLTTSNSYRN-EYYNMNHKYRGKAIIFNHEFFEID--LNPRPGTGKDCENLEKCLKELGFSVDVLSNPK  88

Sf-C1   80 SEEINKFIQQTAEMDHSDADCLLVAVLTHGELGMLYAKDTHYKPDNLWYYFTADKCPTLAGKPKLFFIQACQGDRLDGGITLS------RTETDGSPSTSYRIPVHADFLI 184
Se-C1  108 SDDINKYVQQTADMDHSDADCLLVAVLTHGELGMLYAKDTHYKPENLWYYFTADKCPSLAGKPKLFFIQACQGDKLDGGITLS------RTETDGSPSASYRIPVHADFLI 212
Mb-C1  108 SEEVIKYVQQTADMDHTEFDCLLIAVLTHGELGMLYAKDTHYKPDNLWYYFTGDKCPTLAGKPKLFFIQACQGDKLDGGVNLVS-----YTETDGSPSASYKIPVHADFLI 213
Ha-C1  106 SEEVIRYVQETAEMNHSDFDCLLIAVLTHGELGMLYAKDTHYKPENLWYYFTGDKCPTLAGKPKLFFIQACQGDKLDGGITLS------RTETDGSSSASYKIPVHADFLI 210
Hv-C1  106 SEEVMKYIQETADMDHTDFDCIVIAVLTHGELGMLYAKDTHYKPENLWYYFTGDKCPTLAGKPKLFFIQACQGDKLDGGITLS------RTETDGSSSASYKIPVHADFLI 210
Hs-C1  106 SEEVIKYIQTTADMDHSDFDCLLIAVLTHGELGMLYAKDTHYKPENLWYYFTGDKCPTLAGKPKLFFIQACQGDKLDGGITLS------RTETDGSSSASYKIPVHADFLI 210
Se-C2   79 YTDIMKQIEQTAEMNHSKNDCLLIMVLSHGESGLLYARDTHYRAENLWSPFTDAKCPTLAGKPKLFMFQACQGDKYDDGITLK-----RETETDGWYEP-HKNPVHPDFLL 183
Mb-C2   79 YAEIMKHIKKTAKINHSNHDCLLIVVLIHGEPGKLYAYDTHYRSENLWLRFTEENCPTLAGKPKLFIFQACQGEDYDDGVTLTSSDYIKHIETDGWFEP-DKIPVYPDFLL 188
Ha-C2   88 YGEIMYHIKKTASMNHSKNDCLLIVVLTHGTNGMLYAKDTNYRPEKLWNLFTEENCPTLAGKPKLFVFQACQGEEYDDGITLT-----KNIETDGWFEP-NRNPTHPDFLV 192
Hv-C2   88 YAEIMNHVKKIASMNHSKNDCLLIVVLTHGMNGRLFAKDTDYRPEKLWNPFTAENCPTLAGKPKLFVFQACQGEDYDDGITLT-----KLSETDGWFEP-DRNPTHPDFLV 192
Hs-C2   89 YAEIMNHVKKIASMNHSKNDCLLIVVLTHGMNGKLFAKDTDYRPEKLWGPFTEENCPTLAGKPKLFVFQACQGEDYDDGITLT-----KHSETDGWFEP-DRNPTHPDFLV 193

Sf-C1  185 AFSTVPGYFSWRNTTRGSWFMQALCEELRYAGTE-RDILTLLTFVCQKVALDFESNAPDSAMMHQQKQVPCITSMLTRLLVFGKKQSHL------------------- 272
Se-C1  213 AFSTVPGYFSWRNTTRGSWFMQALCEELRYAGTE-RDILTLLTFVCQRVALDFESNSPDSMIMHQQKQVPCITSMLTRLLVFGKK----------------------- 296
Mb-C1  214 VFSTVPGYYSWRNTTRGSWFMQALCEELRYAGTE-RDILTLLTFVCQRVALDFESNTPDLMPMHQQKQVPCITSMLTRLLVFGKK----------------------- 297
Ha-C1  211 VFSTVPGYYSWRNTTRGSWFMQALCEELRYAGTE-RDILTLLTFVCQRVALDYESNTPDLMPMHQQKQVPCITSMLTRLLVFGRK----------------------- 294
Hv-C1  211 VFSTVPGYYSWRNTTRGSWFMQALCDELRYNGTE-RDILTLLTFVCQRVALDYESNTPDLMPMHQQKQVPCITSMLTRLLVFGRK----------------------- 294
Hs-C1  211 VFSTVPGYYSWRNTTRGSWFMQALCDELRYAGTE-RDILTLLTFVCQRVALDYESNTPDLMPMHQQKQVPCITSMLTRLLVFGRK----------------------- 294
Se-C2  184 VLSTVPGYFAWRNRDRGSWFIQALYEELTYTGEDPIDILTLLTYVCQKVALNFQSETSNDV-TNYKKQVPCISSMLTRRLVLAKKPNGKSSSMDSFLNSFKNCSFNFE- 290
Mb-C2  189 ARSTVPGYFSWRNIDCGSWFIQALCQEINYSATE-MDILTLLTFVNQKVALNYESSTLNAI-SNRKKEVPCFSSMLTRRLVFTEKPD-NLSSTKSLLDYFKECTTKNKN 294
Ha-C2  193 AKSTVPGYYSWRNSTRGSYFITALCEEINYSGKT-TDILTLLTFVCQKVALNFESSTDEDI-SNEKKQVPCISSTLTRRLVFSEKPD-RSKDMNSFVKCFEDNCLC--- 295
Hv-C2  193 AKSSVPGYYSWRNSSRGSWFISALCEELYYSAKT-TDILTLLTFVCQKVALNFESSTLEDD-SNEKKQVPCISSMLTRRLVFSEKPE-SSEDINSLVKCFEDIVL---- 294
Hs-C2  194 AKSSVPGYYSWRNSSRGTWFISSLCEELYYSGKT-TDILTLLTFVCQKVALNFESSTLEDV-SNEKKQVPCISSMLTRRLVFNEKPE-SSQDMNSLVKCFEDNCL---- 295
```
