# Supplementary material for: A comprehensive characterization of the caspase gene family in insects from the order Lepidoptera
Source: BMC Genomics. 2011 Jul 8;12:357. doi: 10.1186/1471-2164-12-357 (PMC3141678; doi:10.1186/1471-2164-12-357)

Figure S8. Phylogenetic relationship between caspase sequences found in Lepidoptera and in Diptera. Amino acid alignments of caspases sequences without their prodomain were used to build a phylogenetic tree generated by the Neighbor joining method (1000 replicates).

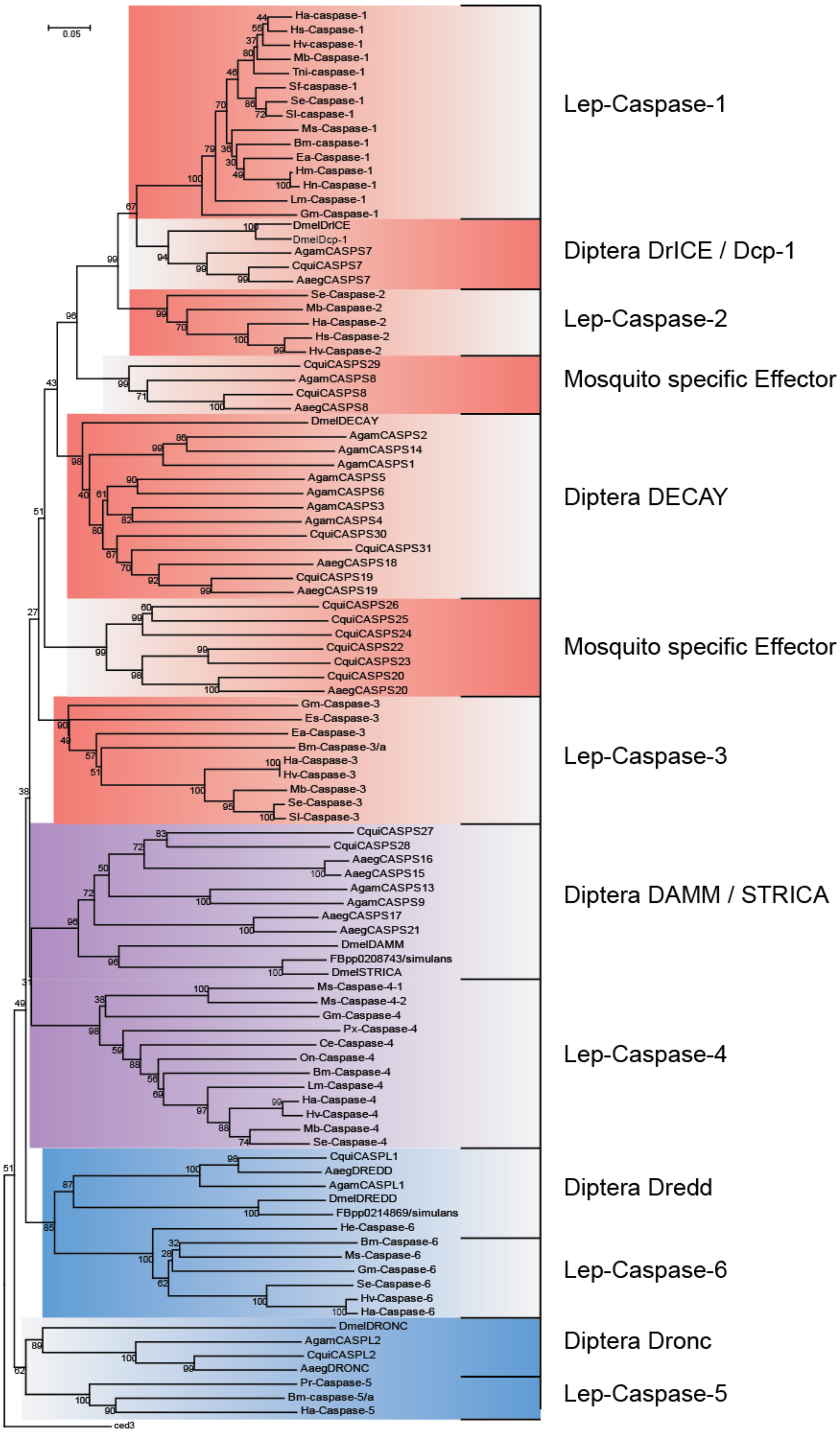

Supplement: Additional file 9 — Figure S8. Phylogenetic relationship of caspases sequences found in Lepidoptera and Diptera. [file 1471-2164-12-357-S9.PDF]
