# Supplementary material for: A comprehensive characterization of the caspase gene family in insects from the order Lepidoptera
Source: BMC Genomics. 2011 Jul 8;12:357. doi: 10.1186/1471-2164-12-357 (PMC3141678; doi:10.1186/1471-2164-12-357)
Supplement: Additional file 10 — Figure S9. Alignment of Bm-Caspase-5 with the prodomains of Drosophila Dronc, Aedes aegypti Ae-Dronc and human Caspase-1, -2 and -9. [file 1471-2164-12-357-S10.PDF]

Figure S9. Alignment of Bm-Caspase-5 with the prodomains of Drosophila Dronc, Aedes aegypti Ae-Dronc, human caspase-1, -2 and -9.
Black boxes outline the predicted α-helices. Each prodomain contain one bundle composed of 6 α-helices.
Secondary structure was predicted using Jpred3 (http://www.compbio.dundee.ac.uk/www-jpred/).

```
Human Casp-9   -------------------------------MDEADRRLLRRCRLRLVEELQVDQLWDAL
Human Casp-2   MAAPSAGSWSTFQHKELMAADRGRRILGVCGMHPHHQETLKKNRVVLAKQLLLSELLEHL
Human Casp-1   MAD--------------------------KVLKEKRKLFIR----SMGEGT-INGLLDEL
Ae-Dronc       -------------------------------MDQRDRHQIQHNMDQLIQHTNYGVLMAEC
Dronc          MQPPELE----------------------IGMPKRHREHIRKNLNILVEWTNYERLAMEC
Bm-Caspase-5   -------------------------------MQEEHKKAIQRNFSSLVEGTDLDSMVMAL

Human Casp-9   LSSELFRPHMIEDIQRAGSGS--------RRDQARQLIIDLETRGSQALPLFISCLEDTG
Human Casp-2   LEKDIITLEMRELIQAKVGS----------FSQNVELLNLLPKRGPQAFDAFCEALRETK
Human Casp-1   LQTRVLNKEEMEKVKRENATV---------MDKTRALIDSVIPKGAQACQICITYICEED
Ae-Dronc       VGRQMMSEVMKAIIEDRYPD---------EASRHKKLFEKITKRGPHAFDILVSICQRNF
Dronc          VQQGILTVQMLRNTQDLNGKPFNMDEKDVRVEQHRRLLLKITQRGPTAYNLLINALRNIN
Bm-Caspase-5   YEKGVFSQQMIEPYRDTSIT---------PRERKRILYREITRRGPQAFTMLLEALREMG

Human Casp-9   QDMLASFLRTNRQAA----LSKPT------------LENLTP-VVLRPEIRK-P------
Human Casp-2   QGHLEDMLLTTLSGL-----QHVLPP------------LSCD-YDL--SLPF-P------
Human Casp-1   S-YLAGTLGLSADQT-----SGNYLN----------MQDSQ--GVL--SSFPAP------
Ae-Dronc       --PTAYKLLKNGNVSTVGSSSRNSSQFEFNSTYHPNRVRSISAGDYSGSELLRQRLANSY
Dronc          C-LDAAVLLESVDE------SDSRPPFISLNERRTSRK---SADIL--SIGS-P------
Bm-Caspase-5   HWDLVRDLDPNSPLQLRAR-----RPVLPP--------------NL--SGPQD-------

Human Casp-9   --EVLRPET-PRPVDIGSGGFGDV------------------------GALESLRGNAD--
Human Casp-2   VCESCPLYKKLRLSTDTVEHSLDNKDGPVCLQVKP-------------CTPEFYQTHFQ--
Human Casp-1   --QAVQDNPAMPTSSGSEGNVKLC------------------------SLEEAQRIWKQ--
Ae-Dronc       SEEDDAKNNNDTVEET---TDKGGKRRLQEFGE---------------KVRSLYKVELSAR
Dronc          V---DTPSPEASEGPC---VSKLRNEPLGALTPYVGVVD-GP------EVKKSKKIHGG--
Bm-Caspase-5   NFVSLTIEKHKPKNNNNDIIKPRPGSDAPV--PPPPVNSSEPVAIPCFHVKESTHFFED--
```
