# Supplementary material for: A comprehensive characterization of the caspase gene family in insects from the order Lepidoptera
Source: BMC Genomics. 2011 Jul 8;12:357. doi: 10.1186/1471-2164-12-357 (PMC3141678; doi:10.1186/1471-2164-12-357)
Supplement: Additional file 11 — Figure S10. Alignment of Bm-Caspase-6 with the prodomains of Drosophila Dredd, Aedes aegypti Ae-Dredd and human Caspase-8 and -10. [file 1471-2164-12-357-S11.PDF]

Figure S10. Alignment of Bm-Caspase-6 with the prodomains of Drosophila Dredd, Aedes aegypti Ae-Dredd, human caspase-8 and -10.
Black boxes outline the predicted α-helices. Each prodomain contain 2 bundles composed of 6 α-helices.
Secondary structure was predicted using Jpred3 (http://www.compbio.dundee.ac.uk/www-jpred/).

```
Human Casp-10   MKSQGQHWYSSSDKNC----KVSFREKLLIIDSNLG--VQDVENLKFLCIGLVPNKKLEK
Human Casp-8    M---------------------DFSRNLYDIGEQLD--SEDLASLKFLSLDYIPQRKQEP
Ae-Dredd        M------------------SISIQLEDLDFIEEELH--FDDKVAVLFLLYG---------
Dredd           M----------AGSNLLIHLDTIDQNDLIYVERDMN--FAQKVGLCFLLYG-------DD
Bm-Caspase-6    MFRPDALDERAALDRQIIHSNLINVDVISQIERELQDEPYDMVSLVFLLY----------

Human Casp-10   SSSASDVFEHLLAE-------------------------DLLSEEDPFFLAELLYII-RQ
Human Casp-8    IKDALMLFQRLQE-------------------------KRMLEESNLSFLKELLFRINRL
Ae-Dredd        HRNPKYVLQLLTA----IRAPDARTQFLSEWANLVAKGEVSASRWQSELLEALAIVQA--
Dredd           HSDATYILQKLLAM----TRSDFPQSDLLIKFA------KSRPETWRRHLVEALCIIGA-
Bm-Caspase-6    -EVPDTALQKLVTHQKIVTEMLGTNLNLLHDWYQH----SKSKPTWKHEFLEALLICQL-

Human Casp-10   KKLLQHLNCTKEEVERLL--PTRQRVS--------------------------------L
Human Casp-8    DLLITYLNTRKEEMERELQTPGRAQISAYRFHFCRMSWAEANSQCQTQSVPFWRRVDHIL
Ae-Dredd        NLCLLKCGFDDEELRDQFL-PHVPELATHVH----------------------------P
Dredd           RKVLRRLGFCWQELRMHYL-PHIAGITLHVH----------------------------P
Bm-Caspase-6    FNIVRRIGFDVQTLRKHYQ-TDYPGLSMYVD----------------------------P

Human Casp-10   FRNLLYELSEGIDSENLKDMIFLLKDS---------LPKT-EMTSLSFLAFLEKQGKIDE
Human Casp-8    IRVMLYQISEEVSRSELRSFKFLLQEEISKCKLDDDMN------LLDIFIEMEKRVILGE
Ae-Dredd        VLKGLWLLCEKMDRATSDRLENYLRQNY-------ALAVMDSEFLEMIMLDMIGQGVIKL
Dredd           LLKSLYRMCEELSLVQSGRLLLDVREKVESQQAGDPLRFYDPAYLEIFLLDWLTRRSIKL
Bm-Caspase-6    LRKILYKICEEIDTPNLIKLQKSLLTY--------DIDVSGHNICEIILLELMSRRFIGI

Human Casp-10   D----------------------------NLTCLEDLCKTVVPKLLRNIEKYKREKAIQI
Human Casp-8    G----------------------------KLDILKRVCAQINKSLLKIINDY--------
Ae-Dredd        GSKEGGQ-------VSDLSNLIAAFKAL-EMEGLKDFCKNIESNFNRDLQSG--------
Dredd           GDINAAG--------SDVQLLVGHLKSNGL-QAQANLLKDT-------IISN--------
Bm-Caspase-6    KYCRHDEKYLTEIKIDKFLRIIENFDGLRKLSLDLKFLQNKFAN-----ETN--------

Human Casp-10   VTPPVDKEAESYQGEEELVSQT----------------DVKTFLE---------------
Human Casp-8    -EEFSKGEELCGVMTISDS---------------PREQDSE-------------------
Ae-Dredd        -ENQSDNAKSTLVAEVKGQPV-----------------DN--------------------
Dredd           APEPDAAGTAAMAVKQEIES------------------DNQQSYC---------------
Bm-Caspase-6    -------PNNSLKCKMDTTSQQNEQPVLVEHTNEGGIFDINNLLDIHEDLEKLQINDGFH
```
