# Supplementary material for: A comprehensive characterization of the caspase gene family in insects from the order Lepidoptera
Source: BMC Genomics. 2011 Jul 8;12:357. doi: 10.1186/1471-2164-12-357 (PMC3141678; doi:10.1186/1471-2164-12-357)

Figure S11: Intron-exon structure of lepidopteran caspases. For each gene, genomic structure is presented first with exons represented by colored boxes and introns by lines. Exon length in bp are indicated under the boxes, intron length above the lines.

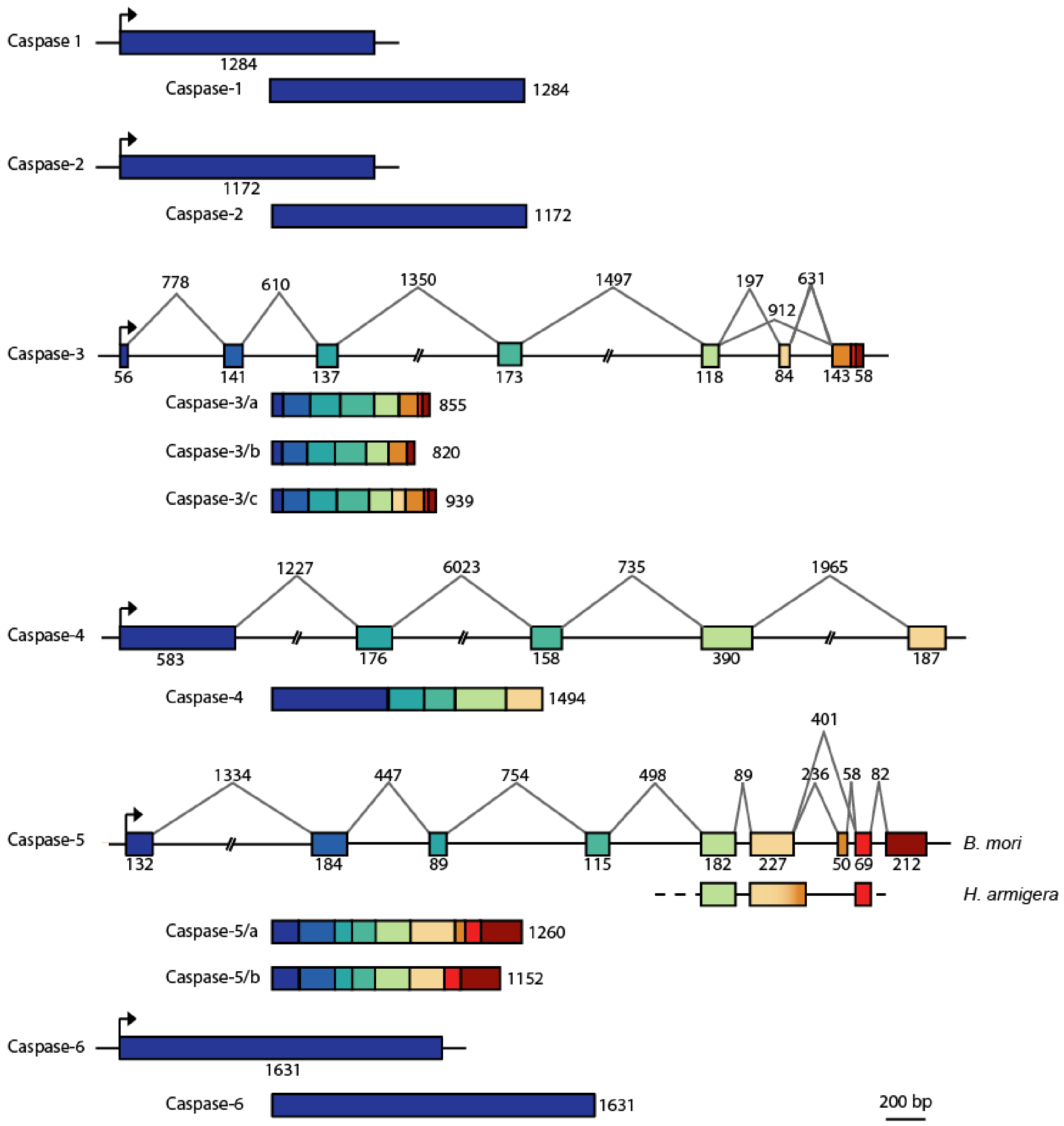

Supplement: Additional file 12 — Figure S11. Intron-exon structure of lepidopteran caspases. [file 1471-2164-12-357-S12.PDF]
