# Supplementary material for: A comprehensive characterization of the caspase gene family in insects from the order Lepidoptera
Source: BMC Genomics. 2011 Jul 8;12:357. doi: 10.1186/1471-2164-12-357 (PMC3141678; doi:10.1186/1471-2164-12-357)
Supplement: Additional file 13 — Figure S12. P-distances of synonymous and non-synonymous substitutions among the Noctuids caspase-1 and -2. [file 1471-2164-12-357-S13.PDF]

**Figure S12**. P-distances of synonymous and non-synonymous substitutions among the Noctuids caspase-1 and -2 sequences using the Nei-Gojobori algorithm with pairwise deletion of gaps and missing data.

Nonsynonymous substitutions per nonsynonymous site ($d_N$)

| | Ha-C1 | Hv-C1 | Hs-C1 | Se-C1 | Mb-C1 | Ha-C2 | Hv-C2 | Hs-C2 | Se-C2 | Mb-C2 |
|---|---|---|---|---|---|---|---|---|---|---|
| Ha-C1 | | 0,030 | 0,020 | 0,060 | 0,040 | 0,300 | 0,310 | 0,310 | 0,290 | 0,300 |
| Hv-C1 | 0,271 | | 0,030 | 0,070 | 0,050 | 0,300 | 0,310 | 0,310 | 0,300 | 0,300 |
| Hs-C1 | 0,260 | 0,140 | | 0,070 | 0,040 | 0,300 | 0,310 | 0,310 | 0,300 | 0,300 |
| Se-C1 | 0,658 | 0,650 | 0,659 | | 0,060 | 0,290 | 0,300 | 0,300 | 0,290 | 0,300 |
| Mb-C1 | 0,532 | 0,538 | 0,528 | 0,564 | | 0,290 | 0,300 | 0,300 | 0,290 | 0,300 |
| Ha-C2 | 0,718 | 0,728 | 0,723 | 0,747 | 0,692 | | 0,080 | 0,070 | 0,220 | 0,220 |
| Hv-C2 | 0,728 | 0,749 | 0,698 | 0,750 | 0,710 | 0,271 | | 0,030 | 0,230 | 0,210 |
| Hs-C2 | 0,701 | 0,776 | 0,738 | 0,788 | 0,748 | 0,260 | 0,175 | | 0,220 | 0,210 |
| Se-C2 | 0,815 | 0,743 | 0,753 | 0,800 | 0,732 | 0,682 | 0,740 | 0,701 | | 0,210 |
| Mb-C2 | 0,786 | 0,783 | 0,804 | 0,806 | 0,765 | 0,628 | 0,688 | 0,670 | 0,680 | |

Synonymous substitutions per Synonymous site ($d_S$)
