# Supplementary material for: A comprehensive characterization of the caspase gene family in insects from the order Lepidoptera
Source: BMC Genomics. 2011 Jul 8;12:357. doi: 10.1186/1471-2164-12-357 (PMC3141678; doi:10.1186/1471-2164-12-357)
Supplement: Additional file 14 — Table S2. Primer sequences used for amplification of caspase transcripts. [file 1471-2164-12-357-S14.PDF]

Table S2. Primer sequences used for amplification of caspase transcripts.

Gene	Primer sequence
Bm-Caspase-4	BmC4R1: 5' CAGCATATCCGCTTCAATTGGC
Bm-Caspase-5	BmC5F1: 5' GATACTAGTATCACACCTCG<br>BmC5R1: 5' CGGGAGGTCCGTGAAGTT<br>BmC5R2: 5' TCACTCGTACAGACCGGGGTG<br>BmC5F3: 5'CACTACAACTGCGAGCCAGA<br>BmC5F4: 5'GTGGACTGCGACAAGTTGAAG<br>BmC5R3: 5'GCACAGGACCTGGATGTACC
Bm-Caspase-6	BmC6F2: 5' TATGATATAGATGTAAGCGGG<br>BmC6R1: 5' ATTTTGGATTTGTTTCATTGGC
Ea-Caspase-1	EaC1RACEF: 5′ TGCCTGTAGACAGGAACGCACCGTTT<br>EaC1RACER: 5′ CGCCATGAGTCAGCACCGCAAT
Ea-Caspase -3	EaC3RACEF: 5′ TGCCAAAAGTGGAACCTGACGCAGT<br>EaC3RACER: 5′ AGGCCTGTCGGCTGCGAAAAGA
Ha-Caspase-1	HaC1F1: 5′ ATGTTGGACGGTGATGTTCAAG<br>HaC1R1: 5′ CTTCCTACCAAACACAAGCAA
Ha-Caspase-2	HaC2F1: 5′ ATGGAAAATATGGACGAAACTT<br>HaC2R1: 5′ AAATTTCAGCAGCTTTGTCAGC
Ha-Caspase-5	HaC5F1: 5' ATGGAGCAGAAACACAAAGAAGC<br>HaC5F2: 5' CACAGACCTGGACTCTGTTG<br>HaC5F3: 5' GTAGACACACTGGGTGAACTG<br>HaC5R3: 5' CTCGATGGTGACGTCACAGG<br>HaC5R5: 5' GTAGTCGATGATGTCCATGAGG
Hv-Caspase-6	HvC6F1: 5' CTGATCGCAAGCAGTTAGACATTG<br>HvC6R1: 5' CAAAGTCACATTGTCTGTAAC
Ms-Caspase-1	MsC1RACEF: 5′ GGCGCTACGCCAAGATGCCTGT<br>MsC1RACER: 5′ TCAGCACAGCGACGAGCAAGCA
Ms Caspase-4-1	MsC4-1F: 5′ ATGGACAGTGAACTTCAGGATAC<br>MsC4-1R: 5′TTAAATTCTCTTAATGTATACAAATTTG
Ms-Caspase-4-2	MsC4-2RACEF: 5′ TCCAAGGGCGTTACCCAAAGAATCTGA<br>MsC4-2RACER: 5′ GCCATGCGTCAGGACCGCAATA
Ms-Caspase-6	MsC6RACEF: 5′ CAGCGTTAAAACAAAGGGCGTAGCTCA
