# Supplementary material for: A comprehensive characterization of the caspase gene family in insects from the order Lepidoptera
Source: BMC Genomics. 2011 Jul 8;12:357. doi: 10.1186/1471-2164-12-357 (PMC3141678; doi:10.1186/1471-2164-12-357)
Supplement: Additional file 2 — Figure S1. Amino acid alignment of Lep-Caspase-1 sequences. [file 1471-2164-12-357-S2.PDF]

Figure S1. Amino acid alignment of Lep-Caspase-1 sequences. Identical residues are boxed in grey. Critical amino acids involved in substrate-binding are boxed in purple. Critical amino acids involved in the active site, including the catalytic cysteine residue, are boxed in red. Blue frames indicate cleavage sites.

```
                           10        20        30        40        50        60        70        80        90       100
                   ....|....|....|....|....|....|....|....|....|....|....|....|....|....|....|....|....|....|....|....|
Aa-Caspase-1   MA-DEVAQNNG----GDREQRP--NGSG--DEGDAWG--SHESSHVRRHARMPVDRNSPYYNMNHKNRGIAFIFNHEHFDIHSLKSRTGTNVDSDNLHKV
Ba-Caspase-1   MS-EVQDNGFSEPSSIEPEQRP--NGGGDGDEGDAWGGGSHESSSARRYARMTVDRNAAYYNMNHKNRGMAIIFNHEHFDIHSLKSRTGTNVDSDNLARV
Bm-Caspase-1   MA-DEEKKTNG----SGTDQRK--NGNE--DEGDAWG--SHGSSQGRRYAKMPVERYAHYYNMNHNNRGMAIIFNHEHFEIHNLKSRTGTNVDSDSLSKV
Ep-Caspase-1   MS-EAQPENNGSGGE---EQRP--NGGG--DEGDAWG--SSDPSFSRIVGRMPVDRNAAYYNMKHKHRGFAVIFNHEHFDVHSLKSRTGTNVDSDSLNKV
Gm-Caspase-1   MSQEANQSNNG-----NDEQRA--NGG-D-DEGDAWG--SHGSVNKPRYAKMPVNRNSPYYKMDHKRRGVAVIFNHEFFDVHSLKPRTGTNVDSDNLARV
Ha-Caspase-1   ML-DGDVQDNG---SVETEQRP--NGGG--DEGDAQG--SHDSSHARRFARMPVDRNAPYYNMNHKYRGMAIIFNHEHFDIHSLKSRTGTNVDSDNLAKV
He-Caspase-1   MS-EVNEQDNG-----ETEQRP--NGGG--DEGDAWG--SHESSSIRHYAKMPVERNAPYYNMNHKNRGIAVIFNHEHFDIHNLKSRTGTNVDCDNLSKV
Hm-Caspase-1   MS-EVNEQDNG-----ETEQRP--NGGG--DEGDAWG--SHESSSIRHYAKMPVERNAPYYNMNHKNRGIAVIFNHEHFDIHNLKSRTGTNVDCDNLSKV
Hn-Caspase-1   MS-EVNEQDNG-----ETEQRP--NGGG--DEGDAWG--SHESSSIRHYAKMPVDRNAPYYNMNHKNXXXAVIFNHEHFDIHNLKSRTGTNVDCDNLSKV
Hs-Caspase-1   ML-DGDVQDNG---SVETEQRT--NGGG--DEGDAQG--SHDSSHSRRLARMPVDRNAPYYNMNHKYRGMAVIFNHEHFDIHSLKSRTGTNVDSDNLSKV
Hv-Caspase-1   ML-DGDVQDNG---SVEAEQRA--NGGG--DEGDAQG--SHNSSHSRRIARMPVDRNAPYYNMNHKYRGIALIFNHEHFDIHSLKSRTGTNVDSDNLFKV
Lm-Caspase-1   ME-D-ESLNNG---VEETEQRPN-GGGG--DEGDASG--SRDSSQGARYARMPVERNDPYYNMKHKYRGMAIIFNHEHFNIHSLKSRTGTNVDCDNLSKV
Mb-Caspase-1   ML-DGEPQDNG---SVDTEQRPNANGGG--DEGDALG--SHESSNARRYARMPVDRNAPYYNMNHKYRGMAIIFNHEHFDIHSLKSRTGTNVDSDNLAKV
Ms-Caspase-1   MS-DEEPQNNG----AQEEQRP--NGGG--DEGDAWG--SHESSRVRRYAKMPVDRNAPYYNMGHKHRGMAIIFNHEHFDIHSLKSRTGTNVDGDNLAKV
Se-Caspase-1   ML-DGK-QDNGNVDSVDTKQRT--NGGG--DEGDALG--SHNSSQPTRYARMPVDRNAPYYNMNHKYRGMAIIFNHEHFDIHSLKSRTGTNVDSDNLSKV
Sf-Caspase-1   ML-DGK-QDNGNVDSVDIKQRT--NGGG--DEGDALG--SNSSSQPNRVARMPVDRNAPYYNMNHKHRGMAIIFNHEHFDIHSLKSRTGTNVDSDNLSKV
Sl-Caspase-1   ML-DGK-QDNGNVDSVDTKQRT--NGGG--DEGDALG--SHNSSQPSRIARMPVDRNAPYYNMNHKHRGMAIIFNHEHFDIHSLKSRTGTNVDSDNLSKV
Tn-Caspase-1   ML-DGESQDNG---FVETEQRP--NGGG--DEGDALG--SHESSQSRRYAKMPVDKNAPYYNMNHKYRGMAIIFNHEHFDIHSLKSRTGTNVDSDNLAKV

                          110       120       130       140       150       160       170       180       190       200
               ....|....|....|....|....|....|....|....|....|....|....|....|....|....|....|....|....|....|....|....|
Aa-Caspase-1   LKGLGFLVTVRHNCKAEEVNHYIQQIADLDHSDHDCLLVAVLTHGELGMLYAK-----------------------------------------------
Ba-Caspase-1   LKGLGFRVTVLVDRKADDVNKYPQQISEMDHTDNDCLLIAVLSHGELGMLYAKGTHYKPDNLFYHFTADRCQTLA-------------------------
Bm-Caspase-1   LRGLGFSVTVLHNLRAEDINRYIQQISEMDHTDNDCLLVAVLSHGELGMLYAKDTHYKPDNLWYYFTADKCPTLAGKPKLFFIQACQGDKLDGGITLS-N
Ep-Caspase-1   LRTLGFRVTVLNNLKFEDVNKYIQDLAAMDHSDSDCLLIAVLSHGELGMLYAKDTHYKPDNLWDYFTADKCPTL--------------------------
Gm-Caspase-1   LKNLGFRVTIFNNLRFHELNARVQEIAEMDYSDDDCLLMAVLTHGEMGILYARDTHYKAENLWYHFTADRCPTLAGKPKLFFIQACQGDRLDGGITLT-T
Ha-Caspase-1   LKGLGFRVTALNNLKSEEVIRYVQETAEMNHSDFDCLLIAVLTHGELGMLYAKDTHYKPENLWYYFTGDKCPTLAGKPKLFFIQACQGDKLDGGITLS-R
He-Caspase-1   LKTLGFRVTILNNLKFEDVNRYLQQVAEMDHTENDCLLMAVLSHGEMGMLYAKDTHYKPDTLWYYFTADKCPTLAGKPKLFFIQACQGDKLDGGITLSSX
Hm-Caspase-1   LKSLGFRVTILNNLKFEDVNRYLQQVAEMDHTENDCLLMAVLSHGEMGMLYAKDTHYKPDTLWYYFTADKCPTLAGKPKLFFIQACQGDKLDGGITLSSR
Hn-Caspase-1   LKSLGFRVTILNNLKFEDVNRYLQQVAEMDHTENDCLLMAVLSHGEMGMLYAKDTHYKPDTLWYYFTADKCPTLAGKPKLFFIQACQGDKLDGGITLSSR
Hs-Caspase-1   LKGLGFRVTVLSNLKSEEVIKYIQTTADMDHSDFDCLLIAVLTHGELGMLYAKDTHYKPENLWYYFTGDKCPTLAGKPKLFFIQACQGDKLDGGITLS-R
Hv-Caspase-1   LKGLGFRVTVLTNSKSEEVMKYIQEVADMDHTDFDCLVIAVLTHGELGMLYAKDTHYKPENLWYYFTGDKCPTLAGKPKLFFIQACQGDKLDGGITLS-R
Lm-Caspase-1   LKGLGFRVTVLNNLTSGEILTYIYQTAEMDHSDFDCLLIATLTHGEMGMLYAKDTHYKPETLWSHFTADKCPTLAGKPKLFFIQACQGDKLDAGITLN-R
Mb-Caspase-1   LKGLGFRVTVLSNLKSEEVIKYVQQTADMDHTEFDCLLIAVLTHGELGMLYAKDTHYKPDNLWYYFTGDKCPTLAGKPKLFFIQACQGDKLDGGVNLVSY
Ms-Caspase-1   LKGLGFRVTMLHNSKAEEINKYIQEIADMDHSENDCLLVAVLTHGELGMLYAKDTHYKPDNLWYYFTADKCPTLAGKPNYFFIQACQGDKLDGGITLTNR
Se-Caspase-1   LKGLGFRVTVLNNLKSDDINKYVQQTADMDHSDADCLLVAVLTHGELGMLYAKDTHYKPENLWYYFTADKCPSLAGKPKLFFIQACQGDKLDGGITLS-R
Sf-Caspase-1   LKTLGFKVTVFPNLKSEEINKFIQQTAEMDHSDADCLLVAVLTHGELGMLYAKDTHYKPDNLWYYFTADKCPTLAGKPKLFFIQACQGDRLDGGITLS-R
Sl-Caspase-1   LKGLGFKVTVFTNLKSEEINKYVQQTADMDHSDADCLLVAVLTHGELGMLYAKDTHYKPENLWYYFTADKCPTLAGKPKLFFIQACQGDKLDGGVTLS-R
Tn-Caspase-1   LKGSGFRVTVLTNLKSEEILKYVQQTADMDHSDSDCLLIAVLTHGELGMLYAKDTHYKPENLWYYFTGDKCPTLAGKPKLFFIQACQGDKSDGGVTLS-R

                          210       220       230       240       250       260       270       280       290       300
                   |....|....|....|....|....|....|....|....|....|....|....|....|....|....|....|....|....|....|....|
Aa-Caspase-1   ----------------------------------------------------------------------------------------------------
Ba-Caspase-1   ----------------------------------------------------------------------------------------------------
Bm-Caspase-1   TETDGSSSSSYRIPIHADFLIVFSTVPGYYSWRNTTRGSWFMQSLCEELRNYGTQRDILTLLTFVCQRVALDFESNTPDITPMH-QQKQVPCINSMLTRL
Ep-Caspase-1   ----------------------------------------------------------------------------------------------------
Gm-Caspase-1   TETDGSSSTQYRIPVHADFLIAFSTVPGYFSWRNTTRGSWFMQALCEELRFYGTERDILQLLTFVNQKVALDYESNTPDVPSMH-QQKQVPCITSMLTRL
Ha-Caspase-1   TETDGSSSASYKIPVHADFLIVFSTVPGYYSWRNTTRGSWFMQALCEELRYAGTERDILTLLTFVCQRVALDYESNTPDLMPMH-QQKQVPCITSMLTRL
He-Caspase-1   TETDGSSS-SYRIPFT---LIS------------------------------------------------------------------------------
Hm-Caspase-1   TETDGSSS-SYRIPIHADFLIVFSTVPGYYSWRNTTRGSWFMQALCEELRYSGTERDILTLLTFVTQKVALDYESNTPDMLTMH-QQKQVPCTNTMLTRL
Hn-Caspase-1   TETDGSSS-SYRIPIQHXFLIVFSTVPGYYSWRNTTRGSWFMQALCEELRYSGTRKRYPDLVNFVTQKVALDYESNTPDMLTMH-QQKQAPCTNTMLTRL
Hs-Caspase-1   TETDGSSSASYKIPVHADFLIVFSTVPGYYSWRNTTRGSWFMQALCDELRYAGTERDILTLLTFVCQRVALDYESNTPDLMPMH-QQKQVPCITSMLTRL
Hv-Caspase-1   TETDGSSSASYKIPVHADFLIVFSTVPGYYSWRNTTRGSWFMQALCDELRYNGTERDILTLLTFVCQRVALDYESNTPDLMPMH-QQKQVPCITSMLTRL
Lm-Caspase-1   TETDGSTSVPYKIPVYADFLIVFSTVPGYYSWRNTTRGSWFMQALCEELRYSGTERDILTLLTFVWQRVALDFESNTPDFLTMHEQQKQVPCIT-MLTRL
Mb-Caspase-1   TETDGSPSASYKIPVHADFLIVFSTVPGYYSWRNTTRGSWFMQALCEELRYAGTERDILTLLTFVCQRVALDFESNTPDLMPMH-QQKQVPCITSMLTRL
Ms-Caspase-1   TETDGLSSASYRIPIHADFLIVFSTVPGYYSWRNTTRGSWFMQALCEELRYTGTERDILTLLTFVCQKVALDFESNTPDLLPMH-QQKQVPCITSMLTRL
Se-Caspase-1   TETDGSPSASYRIPVHADFLIAFSTVPGYFSWRNTTRGSWFMQALCEELRYAGTERDILTLLTFVCQRVALDFESNSPDSMIMH-QQKQVPCITSMLTRL
Sf-Caspase-1   TETDGSPSTSYRIPVHADFLIAFSTVPGYFSWRNTTRGSWFMQALCEELRYAGTERDILTLLTFVCQKVALDFESNAPDSAMMH-QQKQVPCITSMLTRL
Sl-Caspase-1   TETDGSPSASYRIPVHADFLIAFSTVPGYFSWRNTTRGSWFMQALCEELRYAGTERDILTLLTFVCQKVALDFESNAPDSAMMH-QQKQVPCITSMLTRL
Tn-Caspase-1   TETDGSPSTSYKIPVHADFLIVFSTVPGYYSWRNTTRGSWFMQALCEELRYAGTERDILTLLTFVCQRVALDFESNTPDLMPMH-QQKQVPCITSMLTRL

               ....|....
Aa-Caspase-1   ---------
Ba-Caspase-1   ---------
Bm-Caspase-1   LLFGKK---
Ep-Caspase-1   ---------
Gm-Caspase-1   LIFGKK---
Ha-Caspase-1   LVFGRK---
He-Caspase-1   ---------
Hm-Caspase-1   LIFNKKQ--
Hn-Caspase-1   LIFNKK---
Hs-Caspase-1   LVFGRK---
Hv-Caspase-1   LVFGRK---
Lm-Caspase-1   LVFGK----
Mb-Caspase-1   LVFGKK---
Ms-Caspase-1   LVFGKK---
Se-Caspase-1   LVFGKK---
Sf-Caspase-1   LVFGKKQSH
Sl-Caspase-1   LVFGKKQSH
Tn-Caspase-1   LVFGKK---
```
