# Supplementary material for: A comprehensive characterization of the caspase gene family in insects from the order Lepidoptera
Source: BMC Genomics. 2011 Jul 8;12:357. doi: 10.1186/1471-2164-12-357 (PMC3141678; doi:10.1186/1471-2164-12-357)
Supplement: Additional file 3 — Figure S2. Amino acid alignment of Lep-Caspase-2 sequences. [file 1471-2164-12-357-S3.PDF]

**Figure S2.** Amino acid alignment of Lep-Caspase-2 sequences. Identical residues are boxed in grey. Critical amino acids involved in substrate-binding are boxed in purple. Critical amino acids involved in the active site, including the catalytic cysteine residue, are boxed in red. Blue frames indicate putative cleavage sites.

```
                       10        20        30        40        50        60        70        80        90       100
               ....|....|....|....|....|....|....|....|....|....|....|....|....|....|....|....|....|....|....|....|
Ha-Caspase-2  MSEGNGVTEQK-LENTNGTRPKCLPESNSYRNEFYNMNHKYRGKAIICNHEHFEIDLKPRP--GTGKDCENLEKCLNYLGFSVDALSNPKYGEIMYHIKK
Hs-Caspase-2  MSEGNGVTEQKKLKSSNGTSPKCLTTSNSYRNEYYNMNHKYRGKAIIFNHEFFEIDLNPRP--GTGKDCENLEKCLKELGFSVDVLSNPKYAEIMNHVKK
Hv-Caspase-2  MSEGNGVTEQK-LKSSNGTRPKCSTTFNSYRNEYYNMNHKYRGKAIIFNHEYFEIDLNPRP--GTGKDCENLEKCLKDLGFSVDVLCDPKYAEIMNHVKK
Mb-Caspase-2  MAERN-----------EETAPHPGP-CNAYRNEFYNMNYKYRGKALIFNHHHYATALELRPRGGTNKDCESLVECLKQLAFSVDVFCDLKYAEIMKHIKK
Se-Caspase-2  MAHSS----------TNGSQVSCAESFNSYKNEFYDMNHKYRGKAIIFNHDRFDIDIPPRA--GSLKDCDNLEECLTALGFSVDIFHNFKYTDIMKQIEQ

                      110       120       130       140       150       160       170       180       190       200
               ....|....|....|....|....|....|....|....|....|....|....|....|....|....|....|....|....|....|....|....|
Ha-Caspase-2  TASMNHSKNDCLLIVVLTHGTNGMLYAKDTNYRPEKLWNLFTEENCPTLAGKPKLFVFQACQGEEYDDGITLT-----KNIETDGWFEPNRNPTHPDFLV
Hs-Caspase-2  IASMNHSKNDCLLIVVLTHGMNGKLFAKDTDYRPEKLWGPFTEENCPTLAGKPKLFVFQACQGEDYDDGITLT-----KHSETDGWFEPDRNPTHPDFLV
Hv-Caspase-2  IASMNHSKNDCLLIVVLTHGMNGRLFAKDTDYRPEKLWNPFTAENCPTLAGKPKLFVFQACQGEDYDDGITLT-----KLSETDGWFEPDRNPTHPDFLV
Mb-Caspase-2  TAKINHSNHDCLLIVVLIHGEPGKLYAYDTHYRSENLWLRFTEENCPTLAGKPKLFIFQACQGEDYDDGVTLTSSDYIKHIETDGWFEPDKIPVYPDFLL
Se-Caspase-2  TAEMNHSKNDCLLIMVLSHGESGLLYARDTHYRAENLWSPFTDAKCPTLAGKPKLFMFQACQGDKYDDGITLK-----RETETDGWYEPHKNPVHPDFLL

                      210       220       230       240       250       260       270       280       290       300
               ....|....|....|....|....|....|....|....|....|....|....|....|....|....|....|....|....|....|....|....|
Ha-Caspase-2  AKSTVPGYYSWRNSTRGSYFITALCEELNYSGKT-TDILTLLTFVCQKVALNFESSTDEDISNEKKQVPCISSTLTRRLVFSEKPD-RSKDMNSFVKCFE
Hs-Caspase-2  AKSSVPGYYSWRNSSRGTWFISSLCEELYYSGKT-TDILTLLTFVCQKVALNFESSTLEDVSNEKKQVPCISSMLTRRLVFNEKPE-SSQDMNSLVKCFE
Hv-Caspase-2  AKSSVPGYYSWRNSSRGSWFISALCEELYYSAKT-TDILTLLTFVCQKVALNFESSTLEDDSNEKKQVPCISSMLTRRLVFSEKPE-SSEDINSLVKCFE
Mb-Caspase-2  ARSTVPGYFSWRNIDCGSWFIQALCQELNYSATE-MDILTLLTFVNQKVALNYESSTLNAISNRKKEVPCFSSMLTRRLVFTEKPD-NLSSTKSLLDYFK
Se-Caspase-2  VLSTVPGYFAWRNRDRGSWFIQALYEELTYTGEDPIDILTLLTYVCQKVALNFQSETSNDVTNYKKQVPCISSMLTRRLVLAKKPNGKSSSMDSFLNSFK

               ....|...
Ha-Caspase-2  DNCLC---
Hs-Caspase-2  DNCL----
Hv-Caspase-2  DNCL----
Mb-Caspase-2  ECTTKNKN
Se-Caspase-2  NCSFNFE-
```
